# Fossils matter: improved estimates of divergence times in *Pinus* reveal older diversification

**DOI:** 10.1186/s12862-017-0941-z

**Published:** 2017-04-04

**Authors:** Bianca Saladin, Andrew B. Leslie, Rafael O. Wüest, Glenn Litsios, Elena Conti, Nicolas Salamin, Niklaus E. Zimmermann

**Affiliations:** 1 Swiss Federal Research Institute WSL, Birmensdorf, Switzerland; 2 Department of Ecology and Evolutionary Biology, Brown University, Providence, USA; 3 Department of Computational Biology, Biophore building, University of Lausanne, Lausanne, Switzerland; 4 Species, Ecosystems, Landscapes Division, Federal Office for the Environment FOEN, Bern, Switzerland; 5 Department of Systematic and Evolutionary Botany and Botanical Garden, University of Zurich, Zurich, Switzerland; 6 Swiss Institute of Bioinformatics, Quartier Sorge, University of Lausanne, Lausanne, Switzerland

**Keywords:** Fossil calibration, Bayesian clock dating, Molecular clock calibration, Node dating, Fossilized birth-death, Phylogeny, Pines

## Abstract

**Background:**

The taxonomy of pines (genus *Pinus*) is widely accepted and a robust gene tree based on entire plastome sequences exists. However, there is a large discrepancy in estimated divergence times of major pine clades among existing studies, mainly due to differences in fossil placement and dating methods used. We currently lack a dated molecular phylogeny that makes use of the rich pine fossil record, and this study is the first to estimate the divergence dates of pines based on a large number of fossils (21) evenly distributed across all major clades, in combination with applying both node and tip dating methods.

**Results:**

We present a range of molecular phylogenetic trees of *Pinus* generated within a Bayesian framework. We find the origin of crown *Pinus* is likely up to 30 Myr older (Early Cretaceous) than inferred in most previous studies (Late Cretaceous) and propose generally older divergence times for major clades within *Pinus* than previously thought. Our age estimates vary significantly between the different dating approaches, but the results generally agree on older divergence times. We present a revised list of 21 fossils that are suitable to use in dating or comparative analyses of pines.

**Conclusions:**

Reliable estimates of divergence times in pines are essential if we are to link diversification processes and functional adaptation of this genus to geological events or to changing climates. In addition to older divergence times in *Pinus*, our results also indicate that node age estimates in pines depend on dating approaches and the specific fossil sets used, reflecting inherent differences in various dating approaches. The sets of dated phylogenetic trees of pines presented here provide a way to account for uncertainties in age estimations when applying comparative phylogenetic methods.

**Electronic supplementary material:**

The online version of this article (doi:10.1186/s12862-017-0941-z) contains supplementary material, which is available to authorized users.

## Background

The genus *Pinus*, with approximately 115 extant species, is the largest genus of conifers and one of the most widely distributed tree genera in the Northern Hemisphere [1]. Pines are an integral component of many Northern Hemisphere ecosystems, and they have a well-documented, rich fossil record [2] stretching back as much as 130–140 million years [3, 4]. Many studies have focused on this genus, particularly with regard to its phylogenetic relationships [1, 5–10], ecology [11, 12], biogeography [13, 14], and the timing of diversification events [15]. There exists a wealth of molecular, morphological and fossil data on the genus. However, no study has yet made full use of all existing data to generate both a fully resolved phylogenetic tree that includes all extant species and a time calibration of such a tree. Such an extensively dated and comprehensive phylogenetic tree will allow us to fill significant gaps in our understanding of the evolutionary and ecological history in pines [16].

The genus *Pinus* has traditionally been divided into two major clades based on the number of vascular leaf bundles (either one or two bundles, corresponding to subgenera *Strobus* and *Pinus*) [1], and previous studies had not been able to consistently resolve relationships within these major clades. In 2005, Gernandt et al. [5] proposed a new classification based on phylogenetic trees inferred from two chloroplast genes, dividing the pines into two subgenera (*Pinus* and *Strobus*), four sections (sections *Pinus* and *Trifoliae* in subgenus *Pinus* and sections *Parrya* and *Quinquefoliae* in subgenus *Strobus*) and 11 subsections (*Australes, Ponderosae, Contortae, Pinus, Pinaster, Strobus, Krempfianae, Cembroides, Balfourianae* and *Nelsoniae*). Although taxonomically comprehensive and widely accepted, their study relied exclusively on sequences from the *matK* and *rbcL* genes, and was thus unable to resolve relationships within several of the subsections. Subsequent studies have improved phylogenetic resolution, but have mostly focused on specific subclades (e.g. [9, 13, 17, 18]). More recently, Parks et al. [6] analyzed the entire chloroplast genome for 107 pine species, which largely confirmed the structure proposed by Gernandt et al. [5] and provided better resolution for much of the tree. However, despite the detailed chloroplast data and the availability of potential fossil calibration points, comprehensive time-calibrated molecular phylogenetic trees remain lacking.

Sound estimations of divergence times within phylogenetic trees benefit from using many fossils that are evenly distributed across the tree, a strategy that better accounts for rate variation when using relaxed molecular clock models [19–21]. In addition, multiple calibrations can overcome negative effects from errors in dating and placement of single fossils [22]. In the genus *Pinus*, a rich fossil record exists, with the first fossil appearing in the Early Cretaceous [3, 4]. Besides Mesozoic pine fossils [3, 4, 23–26], numerous fossils have been described from the Cenozoic era and were placed within various pine clades [27–32]. Despite a rich fossil record, most recent time calibrations of pine divergences have used very few (usually 1–3) fossils [11, 13, 15, 16, 18] (but see [14]). Some of these fossils are controversial regarding their phylogenetic assignment and age (e.g. the use of *P. belgica* as discussed in [15]), leading to inconsistent age estimates of the origin of pines and divergence times of subsections therein. There remains a great need to include a larger number of carefully evaluated fossil constraints, preferably evenly distributed across all major clades, in order to improve our understanding of pine evolution.

Although Bayesian methods using a relaxed molecular clock are widely accepted for time calibration of molecular trees, there is ongoing debate regarding the best strategy to convert fossil information into calibration information [33–36] and methods are still under development [34]. In the widely and commonly used *node dating* method [termed by 36] (ND*,* hereafter), the geological age of the oldest fossil of a specific clade is transformed into a calibration density (also referred to as prior for divergence times [37] or probabilistic calibration priors [38]) to assign a known age range to the stem node (also referred to as calibrating nodes [39]) of the respective clade in the phylogenetic tree [34]. The probabilistic calibration prior accounts for uncertainties underlying the age of the fossil and the likelihood that the true divergence occurred before its first appearance in the fossil record [19, 37]. However, there is no objective way to define the calibration densities and researchers have used different approaches to define them [19, 37, 38, 40]. Recently, the *fossilized birth-death* (FBD, hereafter) method has been introduced as a new approach for time calibration of molecular phylogenetic trees [41, 42]. This method acknowledges that extant species and fossils are both part of the same evolutionary process [41]. No arbitrary calibration densities on internal nodes need to be defined and FBD allows all fossils to be included as ancestors or extinct tips within a clade (instead of summarizing them into calibration densities assigned to nodes as in ND) [41]. The FBD method therefore overcomes some of the known shortcomings of the ND method (well discussed in the literature [36, 42]) and is considered promising [43]. While FBD has the potential to be widely used in the future [43], only a few studies have directly compared these two dating methods [44, 45]. No conclusion has been reached to date as to whether estimated divergence times are in agreement between the two methods [44–46].

Here, we build the first comprehensive phylogenetic tree of *Pinus* calibrated with a large number of fossils across all major clades, using both the ND and the FBD method. More specifically, our objectives are to: (1) provide a revised and well-supported time-scale for the evolution of major subsections of pines; (2) test the sensitivity of age estimates to different dating methods and fossil sets; and (3) provide a revised list of fossils and their phylogenetic placement within the genus for use in further studies on pine evolution.

**Fig. 1:**
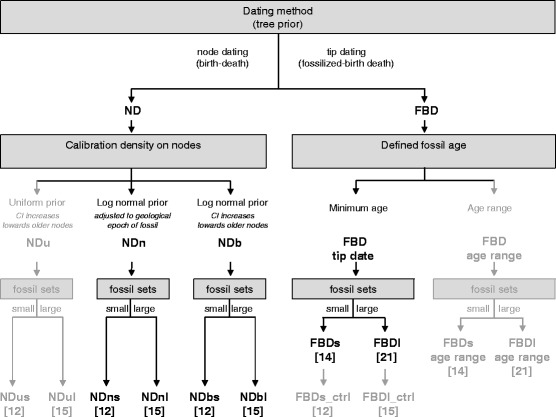
Flow chart illustrating the different dating methods applied. We used both the node dating (ND) and the fossilized birth-death (FBD) method. In ND, we defined the calibration densities on calibration nodes either with narrower (NDn) or broader (NDb) log normal priors on age. NDu is the analog of NDb with uniform priors. In FBD, we defined the fossil age either using a minimum age (FBD tip date) or an age range (FBD age range). Each dating method was carried out with a smaller (s) or larger (l) fossil set (fossil number for each approach indicated in square brackets). Since node dating only uses the oldest fossil per node, this resulted in fewer fossils used in the small and the large fossil set in ND compared to FBD. A control run (FBDs/l_ctrl) was additionally executed for FBD in which exactly the same fossils as in NDs/l were used

To achieve these goals we infer phylogenetic trees based on eight chloroplast sequences within a Bayesian relaxed molecular clock framework using both the FBD and the ND method (Fig. 1). In ND we apply two different approaches for assigning calibration densities on nodes. The first approach follows what was applied in previous pine studies [11, 13, 15] and is presented for comparative purposes only. This approach is based on calibration densities that reflect the geological timescale of the layers in which the fossils were excavated. This approach is problematic because it constrains calibration densities on nodes too tightly, and does not reflect the uncertainty in our prior knowledge (especially toward older nodes). We therefore defined an alternative approach where we constructed calibration densities of increasingly higher uncertainty with increasing age, which better accounts for uncertainty in the a priori information of calibration constraints. In both methods (FBD and ND) we estimated the absolute age scale of the phylogenetic trees from two sets of fossils for each setting (14 or 21 fossils, resp. 12 and 15 in ND due to using only the oldest fossil per node). The two fossil sets differ in our confidence regarding fossil ages and phylogenetic assignments. Our study therefore provides improved estimates of divergence times in pines.

## Results

### Divergence times in *Pinus*

**Fig. 2:**
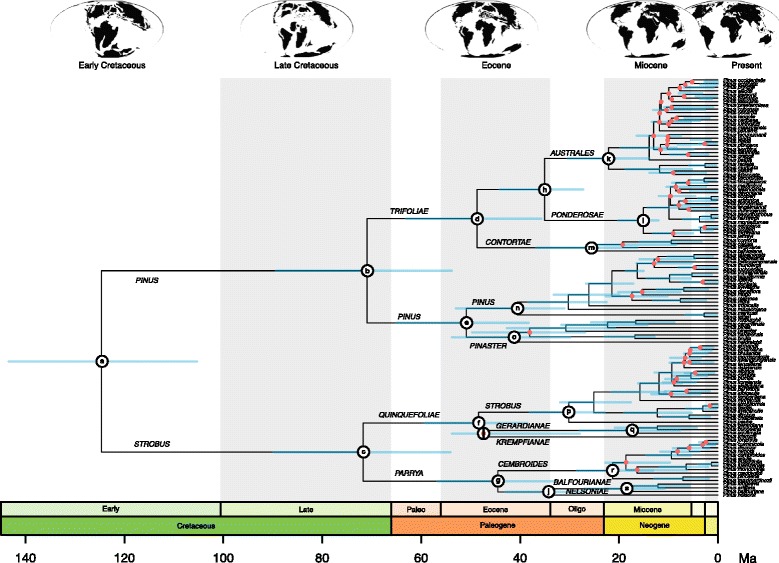
Inferred maximum clade credibility (MCC) tree from results of the FBDl method (fossilized birth-death method, based on the larger set of 21 fossils). Nodes with red dots indicate Bayesian posterior probabilities lower than 0.95, while all other nodes have posterior probabilities higher than 0.95. Light blue lines on nodes represent the 95% highest posterior density (HPD) of the inferred phylogenetic trees. The node labels (*a-s*) indicate those nodes represented in Fig. 3. The geological timescale is in million years and the paleogeographic maps on top were redrawn from [85]

**Fig. 3:**
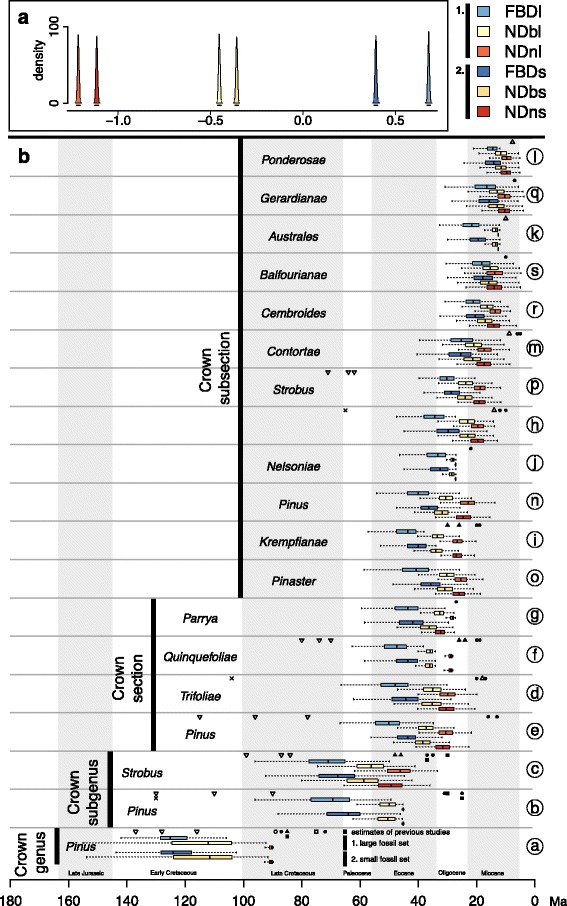
Comparison of estimated node ages of the 19 major clades of *Pinus* across all applied dating approaches. **a**: Densities of effect sizes originate from a mixed-effect model and illustrate to what degree the estimated node ages differ among dating approaches (different colors; see legend) and among fossil sets (1. darker colors for the large, 2. brighter colors for the small fossil set; see legend). The 95% confidence intervals of effect sizes are illustrated with a line below the density curves. Non-overlap of these intervals indicates significant difference on node ages among all 19 nodes. **b**: Boxplots illustrate the estimated node ages across dating approaches and fossil sets for the major clades (*a-s* illustrated in Fig. 2). Whiskers span the 95% highest probability density (HPD), while boxes span the 50% HPD, with the median node age indicated by a vertical bar. The x-axis indicates the geological time in million years. Symbols represent average node ages as estimated in the following studies: Gernandt et al. [16] (filled circle), illustrating estimates resulting from two different calibration scenarios; Hao et al. [13] (filled upward triangles); Willyard et al. [15] (filled squares), illustrating the estimates based on both the chloroplast and the nuclear sequence data, but only presenting results of their 85 Ma calibration scenario as this was indicated to be more realistic; Hernandez-Leon et al. [18] (open upward triangle); He et al. [11] (open circles); Leslie et al. [47] (open squares); Geada Lopez [48] (crosses); Eckert and Hall*.* [14] (open downward triangle). The following abbreviations are used. FBD: fossilized birth-death method; ND: node dating method; l: analyses based on the large fossil set; s: analyses based on the small fossil set; n: narrow calibration priors in ND based on the geological age of the respective fossil; b: broad calibration priors in ND

Our FBD analyses suggest a *Pinus* crown lineage divergence in the Early Cretaceous (node *a* in Fig. 2), irrespective of whether the large fossil set (FBDl: median: 125 Ma, 95% credible interval, CI: 144–106 Ma; Fig. 3) or the small fossil set (FBDs: median: 124 Ma, 95% CI: 145–105 Ma) was used. The estimates from the ND method defined with broader calibration densities on nodes (NDb) support a Late Jurassic to Late Cretaceous age, with the highest probability in the Early Cretaceous, regardless of which fossil set was used. The NDb results favor a slightly younger age for crown *Pinus* than FBD (NDbl: median: 112 Ma, 95% CI: 157 Ma - 95 Ma; NDbs: median: 112 Ma, 95% CI: 160 Ma - 95 Ma). In contrast, the ND approach based on the geological time scale defined with narrow calibration densities (NDn) estimates significantly younger, Late Cretaceous divergence ages with both fossil sets (NDnl and NDns: median: 90 Ma, 95% CI: 96–90 Ma). In FBD, the ages of the crown nodes within the two subgenera (node *b* and *c* in Figs. 2 and 3) are dated similarly to the Late Cretaceous to Eocene (FBDs: median subgenus *Pinus*: 64 Ma, 95% CI: 87–52 Ma; median subgenus *Strobus*: 68 Ma, 95% CI: 89–53 Ma; FBDl: median subgenus *Pinus* 69 Ma, 95% CI: 92–56 Ma; subgenus *Strobus* median: 71 Ma, 95% CI; 92–56 Ma). In the ND method, the major split within subgenus *Strobus* (node *c* in Fig. 2) is dated to the Late Cretaceous to Eocene (NDbs: median: 59 Ma, 95% CI: 79–47 Ma, NDbl: median: 56 Ma, 95% CI: 73–45 Ma; NDns: median: 49 Ma, 95% CI: 63–40 Ma, NDnl: median: 46 Ma, 95% CI: 60–38 Ma). While in ND, the major split within subgenus *Pinus* is dated to the Paleocene to Eocene (NDbs: median: 50 Ma, 95% CI: 63–46 Ma; NDbl: median: 50 Ma, 95% CI: 63–46 Ma; NDns and NDnl: median: 45 Ma, 95% CI: 46–45 Ma). Fig. 3b illustrates the crown age estimates of sectional and subsectional nodes for the methods and approaches used. Maximum clade credibility trees (MCC trees) of all dating methods, approaches and fossil sets are provided in the Additional file 1. Most node age estimates in previous studies are younger than our FBD and NDb results, as well as many of our NDn results (Fig. 3b). One exception is the study of Eckert and Hall [14], who estimated older ages for several deep divergences in *Pinus* which lie outside of our estimated 95% CIs (e.g. crown age of section *Pinus*, *Quinquefoliae*; subsection *Strobus*: node *b, c, e, f, p* in Fig. 3). A number of other node estimates in Eckert and Hall [14] are also older than our corresponding mean posterior ages, although they still overlap with the estimated 95% CI found in this study (e.g. crown age of genus *Pinus* and the crown node of the two subgenera*:* node *a-c* in Fig. 3).

### Comparison of dating methods

The confidence intervals of many of our node age estimates overlap regardless of the method used to estimate them, but posterior mean values are often significantly different among the various techniques. Among the 19 nodes representing crown nodes of subsection and higher-level clades (Fig. 3, nodes *a*-*s*), a few consistent patterns stood out. First, the FBD method estimates significantly older ages than the ND method, irrespective of the specific calibration employed or fossil set used (Fig. 3a). Second, NDn (narrower calibration densities) estimates significantly younger ages than does NDb (broader distribution, Fig. 3a), particularly for the crown age (Fig. 3b). Last, the FBDl analysis (21 fossils) provides significantly older estimates than does the FBDs analysis (14 fossils, Fig. 3a). In both ND methods, in contrast, applying the large set of 15 fossils leads to slightly but significantly younger age estimates than the smaller set (Fig. 3a). Control runs of the FBD method using the same 12 and 15 fossils as in ND reveal very similar node ages as in FBDs (14 fossils) and FBDl (21 fossils) except at the crown node of *Pinus* (see Additional file 2).

### Sensitivity of dating methods to prior settings

We examined the relative influence of the probabilistic calibration priors and sequence data on the Bayesian age estimates in each method (Additional file 3) by comparing the effective prior distributions to the posterior distributions of the calibration nodes. We found significant differences across dating methods: the calibration priors in the NDn method are very similar to the posterior age estimates, revealing the strong influence of the defined calibration priors on estimated node ages. In contrast, the NDb and FBD approaches show increasingly lower influences of the calibration priors, indicating a lower sensitivity of posterior age estimates to calibration priors. This pattern emerges irrespective of the fossil set used (Additional file 3).

### Sensitivity of node age estimates to single fossil exclusions

**Fig. 4:**
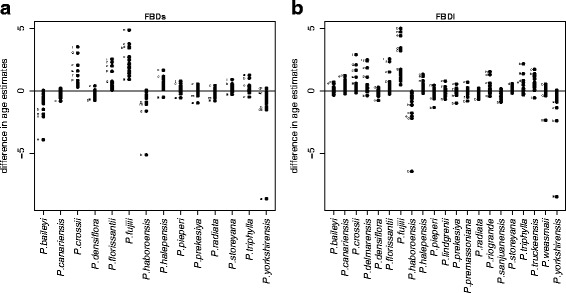
Sensitivity of the time calibration to single fossil exclusion for the fossilized birth-death approaches (FBD). This test measures the difference in age estimates of the 19 major nodes (*a-s*, see also Fig. 3) when keeping versus removing single calibration constraints (fossil, labeled on x-axis) at a time. We illustrate results from the small (**a**) and the large (**b**) fossil set. Letters (see Fig. 2 for assignment) indicate nodes with highest deviations

Figure 4 illustrates how much the 19 nodes (*a-s*) differ in calibrated ages when leaving out the individual fossils in FBDs (Fig. 4a) and FBDl (Fig. 4b; see Additional file 4 for this same sensitivity analysis with the ND-based phylogenetic trees). Including the fossils *P. fujiii* and *P. crossii* in analyses leads to generally younger node ages on almost all 19 nodes compared to analyses where they were left out, both in FBDs and FBDl (Fig. 4) and in NDb approaches (Additional file 4). In NDn approaches, node ages are not sensitive to the exclusion of *P. fujiii,* but they are sensitive to inclusion of the fossils *P. halepensis* and *P. crossii*. In contrast, excluding the oldest fossil (*P. yorkshirensis* in FBD and *P. triphylla* in ND) leads to generally older ages, especially in older nodes. In FBD, a similar pattern is observed for the fossil *P. haboroensis,* while in NDb, excluding *P. baileyi* also leads to older ages (Additional file 4). The exclusion of all other fossils does not have a strong effect on the age estimates of the 19 nodes.

## Discussion

### Divergence times in *Pinus*

The Early Cretaceous crown age of *Pinus* inferred in our study (supported by 95% CI of FBD and 50% CI of NDb, Fig. 3) is approximately 30 Myr older than the age estimated in most previous studies [13, 15, 16, 47, 48] (Fig. 3b). To our knowledge, only one other study estimated a similarly old crown age in *Pinus* [14], but that study incorporated the fossil *P. belgica* [23], which was not used in this study because its exact phylogenetic assignment and age are uncertain [15, 47]. Our estimated crown age is consistent with the recent discovery of the oldest fossil attributed to the genus *Pinus* (*P. mundayi*), which has been dated to the Early Cretaceous (Valanginian, ca. 133–140 Ma) [4] but was not included in our study due to its disputed placement [49]. Although our genus crown age estimates are similar to the one found in the study using *P. belgica*, the crown ages of subgenera and sections inferred in our study are clearly younger than their estimates [14] (Fig. 3b).

In line with the early crown age of the genus *Pinus,* we also found strong evidence for a Late Cretaceous to Eocene origin of the crown of the two *Pinus* subgenera (supported by 95% CI of most dating methods), which is older than the Eocene to Oligocene origin suggested in most previous studies [13, 15, 16, 47]*.* Further, most subsections were thought to have emerged during the Miocene [15], but our results support this conclusion in only few of the subsections (*Ponderosae, Gerardianae, Australes, Balfourianae, Cembroides*). Other subsections date back to the Oligocene or the Eocene as supported by 95% CI, or even in some cases to the Paleocene (in FBDl for subsection *Krempfianae* and *Pinaster*).

The generally older divergence times found in this study have consequences for our understanding of the evolution and biogeographic history of *Pinus*, because splits among important clades may be relevant for understanding changing climates and tectonic configurations (Fig. 2). For example, corridors for high latitude migration became increasingly reduced as the Atlantic Ocean widened and the climate started to fluctuate over the Cenozoic [2], which may have affected the origin and diversification of major clades. The divergence of section *Pinus* and section *Trifoliae* (node b in Fig. 3) may reflect the separation of Laurasia into Eurasia and Laurentia [50], which took place in the Late Cretaceous (~100–66 Ma) [2]. Thus, an age older than ~66 Ma for this node is consistent with this geologic scenario and appears to be reflected in our FBD ages.

Our understanding of the biogeographic history of pines, and its relationship to climatic drivers and geographic constraints, requires accurately dated divergences. This is particularly true for the major crown clades that diversified over the Cenozoic, and whose current diversity has been interpreted to relate to major climatic shifts over the Late Paleogene and Neogene. It is worth noting that although our divergence ages are generally older than those inferred in most previous studies, the majority of extant pine diversity is still estimated to have diverged in the Miocene or later. This may suggest that different drivers were important for the major sectional splits compared to the more recent burst of diversification.

### Possible reasons for older divergence times

Several reasons are likely responsible for the discrepancies in estimated node ages between our study and previous work. Our divergence estimates may differ from other studies because we have used (1) more fossils, (2) more extant taxa, and (3) different model settings, the effects of which we discuss in the following. Dating methods based on only few fossils are very sensitive to the assignment of fossils and defined calibration priors, where assignments can lead to biased substitution rate estimates [39]. If the prior on divergence times derived from a single fossil is inaccurate, then the estimated ages of all nodes will be affected because there are no other calibration points that can mitigate the effects from this error [22, 51]. Even if the single used fossil is accurately placed in the phylogeny, age estimates of nodes distant from the calibration point may still be prone to inaccuracies [35]. The greater number and more even phylogenetic distribution of fossils used in this study is an important step towards a more reliable calibration of the molecular clock, and therefore age estimates, within *Pinus*. Our study also included more extant species than most previous studies, and it has been shown that taxon sampling can have an influence on divergence time inference [52], especially under pronounced lineage rate variation [53]. For example, older age estimates were found with increasing taxon sampling in Malagasy tenrecs [54]. Finally, differences in models themselves relative to other studies may explain our older age estimates. For example, we applied different branching process priors (birth-death and fossilized-birth-death) than previous pine studies (Yule, pure-birth) [11, 13], which has been shown to result in older ages in cycads [43]. Nevertheless, it has also been shown in pines that taxon sampling, clock constraints, the choice of specific sequences, and the selection of silent sites versus all sites had a less pronounced influence on mutation rate estimates (and therefore on divergence estimates) than the choice of fossils and their phylogenetic placement [15].

### Effects on divergence time estimations in our study

In the following paragraphs, we discuss in more detail the differences in age estimates found among the different methods and fossil sets used in this study.

#### Effect of dating method

The reasons for observed differences in age estimates between FBD and ND are potentially manifold, but are primarily due to fundamental differences between the models at the core of FBD and ND methods (see [55] for examples and discussions). The few existing analyses that have compared FBD and ND did not reveal a general trend towards over-or underestimation of node ages [41, 44]. In our study the reason for observing significantly older age estimates in FBD than in ND is primarily due to differences in the placement of fossils within the methods and not to the larger fossil numbers used in FBD. Indeed, FBD runs with exactly the same 12 or 15 fossils as in ND (FBD_ctrl) revealed node ages very similar to those inferred when using the 14 or 21 fossils of the FBD runs presented here (see Additional file 2 for FBD_ctrl comparisons). In ND, we placed fossils at the stem node of the assigned clade, while in FBD we let the same fossil be placed anywhere along the branch descending from the stem node to the crown node, or even anywhere within the crown clade. The minimum age of the stem node in FBD will only be identical to ND if the fossil placement is exactly at the stem node. The farther the fossil is placed away from the stem towards the crown (or even beyond to within the crown clade), the older the minimum age of the stem node will be. When using the “age-range” approach in FBD, the described effect will be even stronger, leading to even older ages, which is what we found (see Additional file 5). While the “tip dating” approach we used for FBD estimates only the minimum possible age range for the divergences, the “age range” approach in FBD reveals the full range of possible node ages, therefore extending these ages further back in time (see Additional file 5).

Erroneous young age estimates in ND are possible if the fossils selected as calibrations do not represent the oldest member of its assigned clade, or if the probabilistic calibration priors, which should correct for this uncertainty, are too narrowly defined. The arbitrary assignment of probabilistic calibration priors is one of the major shortcomings in ND, and age estimates of ND are sensitive to the defined probabilistic calibration priors [19, 56, 57]. No objective approach has yet been suggested to define priors for divergence times, even though it has been shown that incorrect calibration constraints negatively affect divergence estimates [19, 58]. There is a fundamental trade-off between defining priors that are too narrow (which can bias the estimation) or too broad (which may lead to overly large uncertainties), and this sensitivity is visible in our study. First, we found significant differences in the age estimates between NDn and NDb (and NDu, which represents a test in which we used uniform priors), where different density shapes were used (Fig. 3a, resp. Additional file 5 for NDu comparisons). Second, the prior sensitivity analyses (Additional file 3) revealed that posterior age estimates in NDn are significantly more sensitive to the effective priors of calibration constraints than in NDb, whereas FDB is least sensitive. Unless one is certain about narrow prior densities, it seems more conservative to define them broadly and allow for a more balanced influence of both the molecular data and the priors of calibration densities. In our study, we have more confidence in the age estimates of NDb than of NDn, which is consistent with the older divergence times for pines compared to previous studies. The narrow distributions defined in NDn are regarded as problematic as they clearly do not reflect our prior knowledge about paleontological data in pines.

Another known shortcoming in ND is the difficulty in specifying multiple node calibrations, especially when one node is ancestral to another [59], which often occurs when many fossils are used within a clade. As the priors of these multiple constraints interact, the effective prior distributions may be quite different from the initially set prior distributions that were defined based on biological and paleontological knowledge [60]. This is also the case in our study (Additional file 6), where some effective prior distributions of ages were slightly shifted compared to the initially set priors (mainly truncated, as in: *P. premassoniana*, *P. densiflora*, *P. storeyana* in Additional file 6). One of the biggest differences was found in the prior on the calibration node of the fossil *P. premassoniana*. This may reflect the long branch of extant *P. massoniana,* which had probably already emerged during the Oligocene, while the assigned fossil age is younger. The older posterior distribution suggests an older age should be assigned to this node, because the fossil likely does not represent the true age of the divergence in this lineage. Another example where the specified and effective priors differ is in the case of the calibration constraint based on the fossil *P. storeyana*. Here, it is possible that this fossil should be placed in the crown of the “Attenuatae-group” within subsection *Australes* (see discussion Additional file 7C)*.*

#### Effect of fossil choice and assignment

To use a fossil for calibration in the phylogeny, its phylogenetic placement should be unequivocally identified: a task that is not easy given the scarcity of comparable morphological data sets for extinct and extant taxa [61]. In pines in particular, we often encounter the “early but risky” or “safe but late” fossil dilemma [62], where a fossil can either be assigned to a more exclusive clade where its affiliation is doubtful (early but risky), or more conservatively to a more inclusive clade that could reliably contain the fossil but may lead to overly young age estimates (safe but late) [63]. Despite the fact that Gernandt et al. [16] demonstrated that the combination of morphological and molecular data could improve divergence time estimates and phylogenetic relationships within Pinaceae, we lack highly detailed morphological matrices for fossil and extant Pinaceae. Such matrices are rarely available [64, 65] or are difficult to apply to fragmentary plant remains that lack the full richness of morphological and anatomical information of completely reconstructed taxa (e.g. *Pinus arnoldii* Miller [66]). To overcome these problems but still use fossils, we assigned fossils to particular clades based on assumed synapomorphies derived from the distribution of traits among extant taxa. We explicitly tested two different hypotheses in our study: a small fossil set including only “safe” fossils based on traits that we assume to be clear synapomorphies for living clades, and a larger fossil set including additional “risky” fossils where we relaxed the criterion of unambiguous synapomorphies. For the taxonomic assignment of these two sets we relied on the original description and illustrations. Because it is possible that some of these taxonomic assignments are incorrect, we also conducted experiments with alternative hypotheses of fossil placements (see Additional file 7B2). We found that the age estimates inferred in this study were robust against removing the most doubtful fossils (see details in Additional file 7B). The 95% CI of the age distributions of the 19 major nodes (node *a-s*) inferred by these alternative fossil sets overlap the 95% CI of the standard fossil sets used in the primary analyses in most cases (Additional file 7: Figure B2). Exceptions were found at the crown node of the genus *Pinus,* where the 95% CI of NDb without the old fossils (*P. triphylla* and *P. haboroensis*) and without an outgroup (alternative hypothesis 1) resulted in unrealistic old ages. Despite the overlapping 95% intervals between age estimates of different fossil sets, the alternative fossil hypotheses show a tendency towards slightly younger posterior mean age estimates for most of the 19 nodes compared to the primary fossil sets.

#### Which fossil set?

Some calibration points are more inevitably more reliable than others [19], and adding many unreliable fossils could bias estimates of rates and dates. If the addition of the seven “riskier” fossils (those based on a more relaxed criterion of unambiguous synapomorphies) to the smaller fossil set had considerably influenced age estimates, one could expect that the estimated ages would change noticeably when removing these fossils from the dating analyses. We would also expect that this effect would be more severe than when leaving out one of the 14 “conservative” fossils. Including those additional seven fossils indeed led to significantly older (in FBD) or significantly younger (in ND) posterior mean ages, but the differences were inconsistent between the methods as well as fairly small. The 95% credible intervals for most nodes were also overlapping between the small and the large fossil sets. More importantly, the fossils associated with the greatest node age sensitivity (Fig. 4) did not include any of the seven “riskier” fossils.

In summary, our age estimates are robust towards single and multiple changes in the fossil set, and the distribution of the larger fossil set across the phylogenetic tree is defensible, as we find similar results regardless of the fossil set used. Using the larger fossil set allows for the inclusion of all available information to calibrate the relaxed clock models for improved divergence time estimation in pines.

## Conclusions

Our study shows that the divergence time estimations depend on the dating method used, as well as the number of fossils and their phylogenetic placement. Divergence time estimations are dependent on different assumptions inherent in the dating analyses, but are especially affected by the phylogenetic placement of fossils. We urge that future studies relying on dated phylogenetic hypotheses of pines embrace the uncertainty stemming from different calibration approaches, and that the implicit assumptions between dating approaches are considered. This will increase the robustness and confidence in tested hypotheses and improve our understanding of trait evolutionary processes and their ecological and evolutionary implications.

## Methods

### Taxonomy

We used 115 pine species in this analysis (based on availability in GenBank), of which 105 taxa are treated as species by Farjon [67] while 10 additional species used in this study that were treated as synonyms or varieties by Farjon [67]: *P. discolor, P. johannis, P. juarezensis, P. chiapensis, P. kwangtungensis, P. fragilissima, P. cooperi, P. washoensis, P. yecorensis, and P. maestrensis.* Four species included in the taxonomic treatment of Farjon [67] are missing in this study: *P. luzmariae, P. henryi, P. uncinata, and P. wangii.*

### DNA sequence matrices

We downloaded eight plastid gene sequences available in GenBank: *matK* (sampled for 113 taxa, length of 1380 bp in our dataset)*, rbcL* (113 taxa, 1254 bp)*, trnV* (106 taxa, 482 bp)*, ycf1* (101 taxa, 1019 bp)*, accD* (97 taxa, 910 bp)*, rpl20* (95 taxa, 95 bp)*, rpoB* (103 taxa, 343 bp) and *rpoC1* (99 taxa, 383 bp) (Additional file 8). We used the sequences provided in Parks et al. [6] where possible, supplemented with other sequences from Genbank [5, 14, 18, 68–72], some of which are not linked to a published journal (see Additional file 8). We ran an automated alignment for all sequences of each gene using MAFFTv7.1 [73], manually checked it, and removed ambiguously aligned nucleotides using Gblocks with default settings [74]. The concatenated sequences resulted in a matrix consisting of 115 species and a length of 5866 nucleotides. Eighty-five taxa covered all 8 gene sequences (8/8 of genes), 13 taxa covered 7/8, 1 taxon 6/8, 2 taxa 5/8, 4 taxa 4/8, 5 taxa 3/8, 4 taxa 2/8 and 1 taxon 1/8. The coverage in nucleotides was 100% of all bp in 85 taxa, >80% of bp in 99 taxa, >50% of bp in 11 taxa, and only 5 taxa had <50% of bp (*P. bhutanica, P. cooperi, P. jaliscana, P. kesiya, and P. tecunumanii*). We provide all details for the full sequence matrix in the Additional file 9.

### Fossil sets and taxonomic assignment

We selected the fossils according to the following three criteria: (a) the fossil locality could be assigned a precise age; (b) the fossil could be placed to a particular node based on morphological characters; and (c) the selected fossils are distributed evenly across all major pine clades. In this study, we focused on fossils of ovulate cones (except for *P. triphylla*, see Additional file 7C) because other fossil remains (leaves, pollen cones, pollen) are either not commonly described or lack the characters relevant to distinguish clades. We provide more details on these characters in Additional file 7C, and on the age and placement of each fossil in Additional file 7.

We linked fossils to extant taxa based on assumed synapomorphies deduced from the distribution of derived traits among extant species. As these assignments represent a hypothesis of how fossils relate to extant taxa, we tested two different fossil calibration schemes that serve as two different hypotheses of fossils age constraints. The first set (the “short list”) consists of 14 fossil taxa that either exhibit traits we consider unambiguous synapomorphies, or fossil taxa that are morphologically indistinguishable from extant species. Such a conservative approach may bias an analysis towards inferring too young ages, however, because older and often more ambiguous fossil taxa are not considered. The second set (the “long list”) uses a larger number of fossil taxa (21 fossils) and in some cases relaxes the criterion of unambiguous synapomorphies. Analyses of these fossil sets can be directly compared for consistency, and we believe they provide a reasonable bracket on ages within *Pinus*. We further tested the effect of alternative hypotheses (AH) by removing the following fossils from some of the analyses: *P. delmarensis, P. fujiii, P. haboroensis, P. pieperi, P. prekesiya, P. premassoniana, P. riogrande, P. sanjuanensis, P. triphylla, P. truckeensis,* and *P. weasmaii* (see details Additional file 7B).

### Phylogenetic reconstruction

We conducted all analyses with BEAST v2.3.1 [75] and constructed the required input-file using BEAUti 2.3.1 [75] with settings described in detail below. We provide all BEAST input files in the Dryad Digital Repository [76].

#### Partitions, substitution and clock models

We defined the partitions and site models in BEAUti based on the partition scheme and models proposed by PartitionFinder 1.1 [77] (Additional file 10). We applied PartitionFinder using linked branch lengths and the g*reedy* algorithm to search, based on Bayesian Information Criterion (BIC), for the statistically best-fit partitioning schemes and models of nucleotide substitution available in BEAST [75]. Because all gene sequences are from the chloroplast genome and can therefore be expected to be linked, we used the same time-tree for all gene sequences. We further partitioned the clock model and used a separate clock model for the gene sequence *ycf1*, as this gene sequence differed considerably from the others regarding the rate of substitution between lineages, demonstrated in [68]. We checked this for our data set by visually comparing the branch lengths of lineages between inferred single gene trees estimated for each gene sequence separately in BEAST2 (without calibration constraints). Branches between lineages in the *ycf1* gene tree were longer compared to the branches in the other gene trees, while the latter were more similar among each other compared to the *ycf1* gene tree. For all remaining gene sequences we linked the clock models, as they did not differ much among each other regarding their rate of evolution between lineages. For both clock partitions, we used an uncorrelated relaxed molecular clock model with a log-normal prior.

#### Calibration priors to date the phylogenetic trees

To get estimates for the divergence times in *Pinus,* we used different priors on divergence time to calibrate the molecular clock to an absolute timescale (Additional file 7: Table A2). Basically, we applied the node dating method with a birth-death tree prior (ND) and varying node calibration constraints (see details below) and the tip dating method with a fossilized birth-death tree prior (FBD) [41, 42], both implemented in BEAST2 [75].

The ND method uses the age of the oldest fossil within a specific clade as a minimum age constraint for the node at which the clade, including the fossil, had diverged (calibrating node). We defined these calibrating nodes by determining a monophyletic subset of all the taxa belonging to this clade, so called *taxon sets* (see Additional file 7A, Fig. A1). In clades with low phylogenetic resolution, we defined the calibration nodes (monophyletic taxon sets) following the classification in the gene tree of Parks et al. [6]. For the ND method, a prior calibration density is defined at each calibration node to account for uncertainty underlying the age of the fossil and the possibility that the true divergence occurred earlier than defined by this fossil record [34]. To compare our analyses with previous studies on pines and to evaluate the sensitivity of ND analyses to prior calibration densities, we used two different approaches to assign prior calibration densities in the ND analyses. In both ND approaches, we used a log-normal distribution for the calibration density at each calibration node, but we varied the shape and breadth of the log-normal distributions.

In the first approach (NDn), we defined a prior calibration density on the calibrating nodes according to the age range of the geological Epoch in which the respective fossil was found (Additional file 7A, Table A1). This procedure is commonly used in studies of pines [13, 15]. The offset of the log-normal distribution was set to the minimum age of the corresponding Epoch, whereas the 95th quantile represented the maximum age of the Epoch (Additional file 7A, Table A2). In the second approach (NDb), we employed a novel procedure for designing the prior calibration density by systematically varying the parameters for the log-normal distribution by fossil age. Specifically, we assumed that the confidence interval (CI) of the priors is narrow for young nodes (5 Ma for the youngest) and higher for the oldest fossils. We increased the 95th quantile every 5 Ma by 10% of the previous 5 Ma age class, resulting in a 95th quantile of 28 Ma for the oldest (90 Ma) fossil. Hence, we fixed the 95th quantile for the youngest fossil to 5 Ma and for the oldest fossil to 28 Ma, then linearly scaled the s.d. of the log-normal priors between 1.0 (youngest fossil) and 0.6 (oldest fossil). This procedure leads to higher densities of young ages close to the minimum fossil age in the calibration priors of the youngest fossils (strong skew), while the prior densities for the oldest fossils are less skewed and their CI spans a broader range of ages (Additional file 7A). In this second approach, we set the offset of the log-normal distribution to the minimum age derived from the original publications of the fossils (Additional file 7A, Table A2). A more conservative approach of defining calibration densities on calibration nodes in ND would be a uniform distribution, and we provide results for such an analysis in the Additional file 5. We ran each of the prior settings for both sets of fossils, although we could not include all of the listed fossils (Additional file 7) in ND because our technique can only use the oldest fossil of a given clade. We also did not include *P. truckeensis, P. riogrande* and *P. weasmaii* in ND analyses, as it is difficult to justify their node placement without credible synapomorphies. The analyses using the small set (NDns and NDbs) included therefore 12 fossils while analyses using the larger set (NDnl and NDbl) included 15 fossils (Additional file 7A).

In contrast to the ND method, the FBD method treats fossils as members of the assigned clade and the fossils represent sampled ancestors or extinct sister taxa. In this study, the placement of the fossils is constrained by assigning them to their corresponding clade through user-defined monophyletic taxon sets (as illustrated in Additional file 7A). Fossils representing stem members of a certain clade were forced to be placed anywhere along the stem branch of the clade, either as extinct sister tips or direct ancestors (fossils illustrated as black dots in Additional file 7A, Figure A1). Other fossils were assigned as extinct members of a certain clade without knowing their exact placement. In this case, FBD could place these fossils anywhere within the user-defined taxon set, either as ancestor or extinct taxa of any of the branches within the assigned clade (fossils illustrated as red circles in Additional file 7A, Figure A2). FBD does therefore not require specification of calibration nodes and calibration densities to infer absolute ages. Rather, it includes absolute dates (so called *tip dates*) for extant and extinct taxa or a defined range of dates for a fossil in which the MCMC will sample the fossil uniformly. Here, we provide the results of both approaches, the “tip date” and the “age range” approach. In the “tip date” approach, we fixed the ages to absolute dates and defined the tip dates as the number of years before the present the specific taxon was living (fossil dates were based on minimum ages listed in Additional file 7A, Table A2). In the “age range” approach, we defined age ranges for each fossil following the same concept as in NDb except for the oldest fossil (*P. yorkshirensis*) (Additional file 7A, Table A2). This fossil was not used in NDb and would have yielded in an unlikely age range of 185 Ma to 129 Ma. The crown node of *Pinus* is likely not older than 160 Ma [47]. We therefore set an age range spanning from 160 Ma to 129 Ma for *P. yorkshirensis*. We provide the results of this approach in the Additional file 5. As FBD does not require placing fossil constraints to nodes, we could use all of the 14 (FBDs) or 21 (FBDl) fossils from the two fossil sets in the FBD approaches (Additional file 7A, Table A1). We additionally ran control analyses (FBDs_ctrl and FBDl_ctrl) with exactly the same fossils (12 and 15) as in ND, to allow for a direct comparison between the two methods. Further, FBD analyses were based on rho sampling and not conditioned on root sampling since the fossil *P. yorkshirensis* is placed along the stem of *Pinus* and the root node represents a sampled node.

### Posterior analysis and summarizing trees

For each setting, we ran two independent analyses in BEAST for either 3 × 10^8^ or 2 × 10^8^ generations (we found that 2 × 10^8^ generations is more than sufficient to reach convergence and therefore we adjusted some follow up runs to save computational time). We then evaluated the convergence and mixing of the MCMC chains in Tracer v1.6 [75], ensuring that the multiple runs converged on the same distribution and ascertained that effective sample sizes (ESS) exceeded 200. We further compared the effective prior and posterior distributions of all the parameters to test whether our analyses are prior-sensitive and whether the data are informative for the MCMC analyses. We then resampled the resulting files of the inferred phylogenetic trees with a frequency of 10^5^ in logCombiner v2.3.1 [75] and a burn-in of 30% (resp. 46% for the 3 × 10^8^ generation runs). This resulted finally in 1401 (resp. 1411) subsampled trees. In ND, we summarized the subsampled trees with a maximum clade credibility tree with common ancestor heights as node heights using TreeAnnotator v2.3.1 [75]. Because we did not provide morphological character data for the fossils, and single resulting placements within the assigned clades do not represent real resolved relationships within FBD analyses (rather, random placements), we pruned off all fossil lineages in all subsampled trees using the *Full2ExtantConvertor.jar,* written by Alexandra Gavryushkina [78]. We summarized these pruned, subsampled FBD trees the same way as in ND*.*

The posterior age estimates of the subsampled phylogenetic trees of all methods are summarized for 19 selected nodes (node *a-s*) that represent the crown nodes of all major sections in *Pinus*. To test if estimated ages across nodes significantly differ among the methods, we standardized the log-transformed age estimates of every node by first subtracting the mean age across all subsampled trees and methods of that node and second by dividing all ages by the standard deviation of node ages across all subsampled trees and methods of the same node. This yielded overall estimated node-ages across trees and methods with a mean of 0 and a standard deviation of 1 for each node. The resulting age differences were then directly comparable across all nodes, and allowed for estimating the general node-age differences from the overall mean depending on the choice of method and setting (represented as standardized effect size). For this analysis, we used a linear mixed effect model (MCMCglmm [79]) with tree identity as a random effect to account for the inter-dependence of nodes within each of the subsampled posterior trees.

### Prior sensitivity

Priors of multiple calibration constraints can interact and may lead to joint effects, especially when one constraint is ancestral to the other [59]. This is a major shortcoming of ND. We therefore tested if our initial priors are similar to the effective priors. For this we ran all MCMC analyses without any sequence data to sample only from the prior distribution as recommended by [38], and results illustrated in Additional file 6. In addition, we compared the effective prior calibration densities with the posterior calibration densities to examine the relative influence of the prior and the sequence data on the age estimates [38]. We illustrated this comparison in a figure by plotting the effective prior against the posterior distribution for both the two ND approaches and the FBD approaches for the small and the large fossil set. For FBD, we illustrated the most recent common ancestral node of the clade the respective fossil was assigned to. We tested whether the methods are significantly more or less sensitive to the priors on time by applying a paired Wilcox test.

### Sensitivity of calibration approaches to single fossil exclusion

To test whether the results are sensitive to the removal of individual fossils we analyzed to what degree the age estimates of the 19 major nodes change in response to removing one single calibration constraint at a time. To do so, we first sampled the 19 node ages from each of the subsampled trees within each analysis when all fossils were used. Next, we ran specific analyses for each method and fossil set, by iteratively leaving out fossils as a calibration constraint, one at a time. Finally, we illustrated the differences in node ages in response to keeping versus removing one single calibration constraint at a time (see Additional file 4 for ND). Note, for the FBD method the most recent common ancestor of the clade a fossil belongs to is represented. These analyses were carried out for both fossil sets.

### Statistical analyses

All statistical analyses and illustrations were generated in the statistical computing environment *R* [80] using the packages phyloch [81], ape [82], geiger [83], raster [84], and MCMCglmm [79]*.*

## Additional files

Additional file 1: Additional maximum clade credibility (MCC) trees. The trees originate from the node dating (ND) method with narrow prior calibration densities (NDn), with broad prior calibration densities (NDb), and from the fossilized birth-death (FBD) method. Each of these three methods was used in combination of either a small (s) or a large (l) fossil set. The following MCC trees are shown: (A) NDns, (B) NDnl, (C) NDbs, (D) NDbl, (E) FBDs. Note that the MCC tree for FBDl is given in Fig. 2. Nodes with red dots indicate Bayesian posterior probabilities lower than 0.95, while all other nodes have posterior probabilities higher than 0.95. Light blue lines on nodes represent the 95% highest posterior density (HPD) of the inferred phylogenetic trees. The node labels (*a-s*) indicate those nodes represented in Fig. 3. The geological timescale is in million years. (PDF 204 kb)

Additional file 2: Comparison of estimated node ages of the 19 major clades of *Pinus* across all applied dating approaches. **A**: Densities of effect sizes originate from a mixed-effect model and illustrate to what degree the estimated node ages differ among dating approaches (different colors; see legend) and among fossil sets (1. darker colors for the large, 2. brighter colors for the small fossil set; see legend). The 95% confidence intervals of effect sizes are illustrated with a line below the density curves. Non-overlap of these intervals indicates significant difference on node ages among all 19 nodes. **B**: Boxplots illustrate the estimated node ages across dating approaches and fossil sets for the major clades (*a-s* illustrated in Fig. 2). Whiskers span the 95% highest probability density (HPD), while boxes span the 50% HPD, with the median node age indicated by a vertical bar. The x-axis indicates the geological time in million years. The following abbreviations are used. FBD: fossilized birth-death method; ND: node dating method; l: analyses based on the large fossil set; s: analyses based on the small fossil set; n: narrow calibration priors in ND based on the geological age of the respective fossil; b: broad calibration priors in ND. (PDF 116 kb)

Additional file 3: Comparison of the effective age prior density against the posterior calibration densities (Bayesian phylogenetic age estimate) for the three dating approaches used (FBD: fossilized birth-death (blue); NDn/b: node dating with narrow (red) and broad (orange) prior distributions; s/l: small (**A**) and large (**B**) fossil sets. Boxplots represent the absolute deviation from the 1:1 line, while letters indicate significant differences in absolute deviations at the level of *p* = 0.05 (based on a paired Wilcox test). (PDF 116 kb)

Additional file 4: Sensitivity of the time calibration to single fossil exclusion for the node dating approaches (ND). This test measures the difference in age estimates of the 19 major nodes (*a-s*, see also Fig. 2) when keeping versus removing single calibration constraints (fossil, labeled on x-axis) at a time. NDns and NDnl are based on narrow prior calibration densities using the small (**A**) and the large (**B**) fossil set, respectively. NDbs and NDbl are based on broad prior calibration densities using the small (**C**) and the large (**D**) fossil set, respectively. Letters (see Fig. 2 for assignment) indicate nodes with highest deviations. (PDF 125 kb)

Additional file 5: Comparison of estimated node ages of the 19 major clades of *Pinus* across all applied dating approaches. Boxplots illustrate the estimated node ages across dating approaches and fossil sets for the major clades (*a-s* illustrated in Fig. 2 **)**. Whiskers span the 95% highest probability density (HPD), while boxes span the 50% HPD, with the median node age indicated by a vertical bar. The x-axis indicates the geological time in million years. The following abbreviations are used. FBD: fossilized birth-death method using “tip date” approach. FBD age range using “age range” approach. ND: node dating method; l: analyses based on the large fossil set; s: analyses based on the small fossil set; n: narrow log normal calibration priors in ND based on the geological age of the respective fossil; b: broad log normal calibration priors in ND; u: uniform calibration priors. The lower and upper limits of the uniform distribution represent the 2.5% and 97.5% CI levels used for the log-normal priors in the NDb method. (PDF 68 kb)

Additional file 6: Comparison of the specified calibration prior in BEAUti (log-transformed) against the effective calibration prior (log-transformed), as estimated without sequence data, and illustrated for all node dating approaches (ND). NDns and NDnl are based on narrow prior calibration densities using the small (**A**) and the large (**B**) fossil set, respectively. NDbs and NDbl are based on broad prior calibration densities using the small (**C**) and the large (**D**) fossil set, respectively. Labels are only given for fossil constraints with high deviance from the 1:1 line. (PDF 69 kb)

Additional file 7: Overview of fossils used in this study. The following abbreviations and symbols are used: FBD: fossilized birth-death method; ND: node dating method; l: denotes analyses based on the large fossil set; s: denotes analyses based on the small fossil set; n: narrow calibration priors in ND based on the geological age of the respective fossil; b: broad calibration priors in ND. “x” in tables indicates which fossil was used in the different fossil sets. Asterisks on fossil numbers represent those used in the large fossil set only. **A:** the fossils used for calibration constraints are listed with the minimum age of each fossil listed. Table A1: Summary of fossils used in each fossil set and the geological layer (and the time scale thereof in Ma) in which the fossils were excavated with the corresponding references. Table A2: Specified prior calibration densities used for Bayesian clock methods. Fig. A1: Illustration of taxonomic assignment for each fossil in ND. Fig. A2: Illustration of taxonomic assignments for each fossil in FBD. Red dots indicate fossils that were allowed to be placed anywhere within the corresponding clade. Black dots illustrate fossils that were allowed to be placed anywhere along the indicated branch. **B:** Descriptions of alternative hypotheses (AH). Table A3: Summary of fossils used in each fossil set of the alternative hypotheses (AH). Fig. A3: Illustration of taxonomic assignment of fossils in the four alternative hypotheses. Fig. A4: Estimated ages by each of the four alternative hypotheses compared to the primary age estimates in Fig. 3. Boxplots illustrate the estimated node ages across dating approaches and fossil sets for the major clades (*a-s* illustrated in Fig. 2). Whiskers span the 95% highest probability density (HPD), while boxes span the 50% HPD, with the median node age indicated by a vertical bar. The x-axis indicates the geological time in million years. **C:** Fossil descriptions and justifications ordered by taxonomic groups. Bold-italic font represents those fossils used in the smaller fossil set, while italic font represents those used in the larger fossil set only. (PDF 508 kb)

Additional file 8: Accession numbers of used gene sequences downloaded from GenBank. Asterisks on accession numbers indicate sequences that are not linked to a published journal article. (PDF 67 kb)

Additional file 9: Sequence matrix used for phylogenetic inference. The number of nucleotide base pairs per gene sequence used for each pine species. N in parentheses gives the number of positions in this gene sequence for which the nucleotide pair is undetermined. (PDF 68 kb)

Additional file 10: Settings and output summary of PartitionFinder showing the best partition scheme for the used sequence data in this study. (PDF 30 kb)
