# Supplementary figures and images for: Fossils matter: improved estimates of divergence times in Pinus reveal older diversification
Source: BMC Evol Biol. 2017 Apr 4;17:95. doi: 10.1186/s12862-017-0941-z (PMC5381128; doi:10.1186/s12862-017-0941-z)

A.

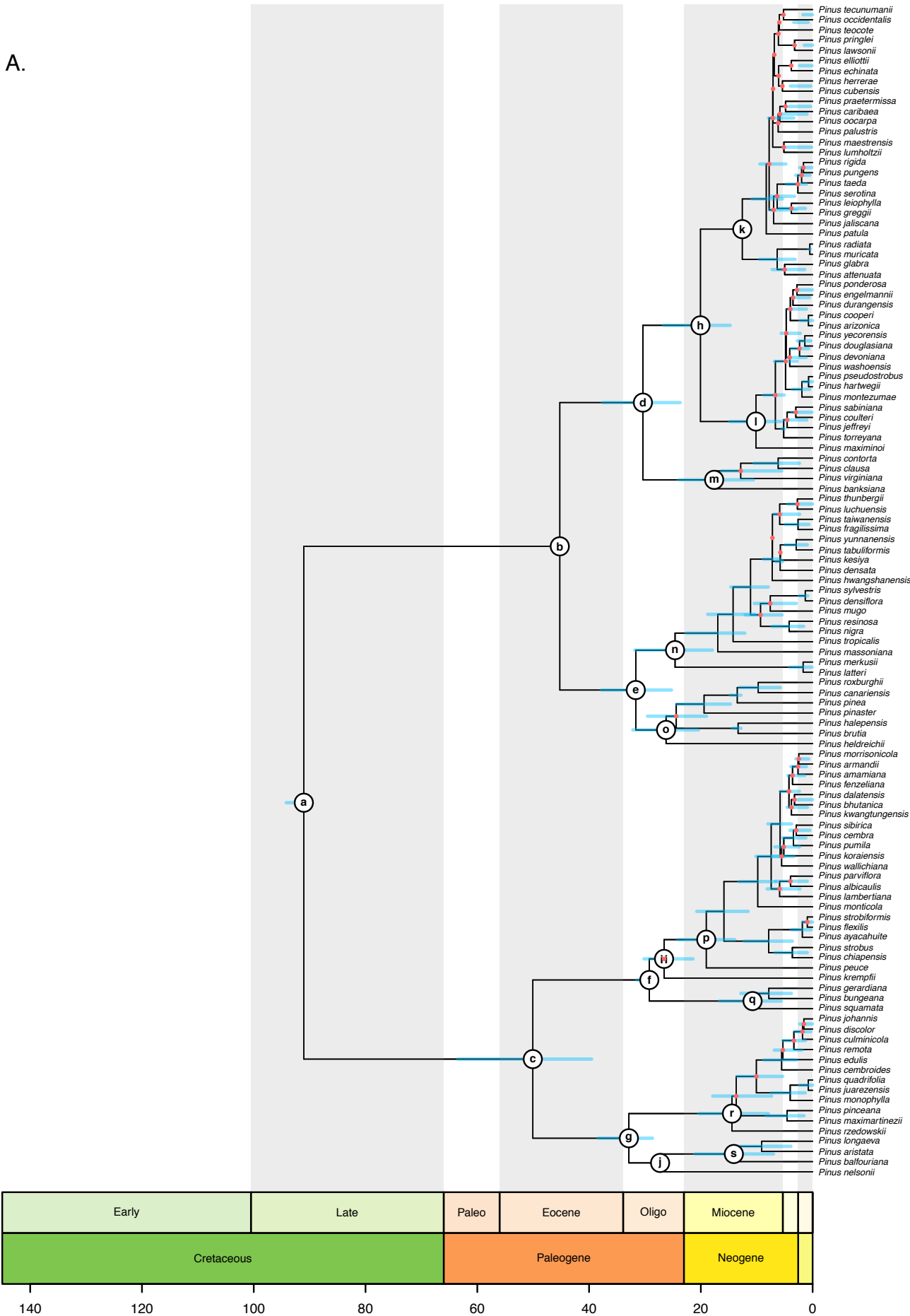

B.

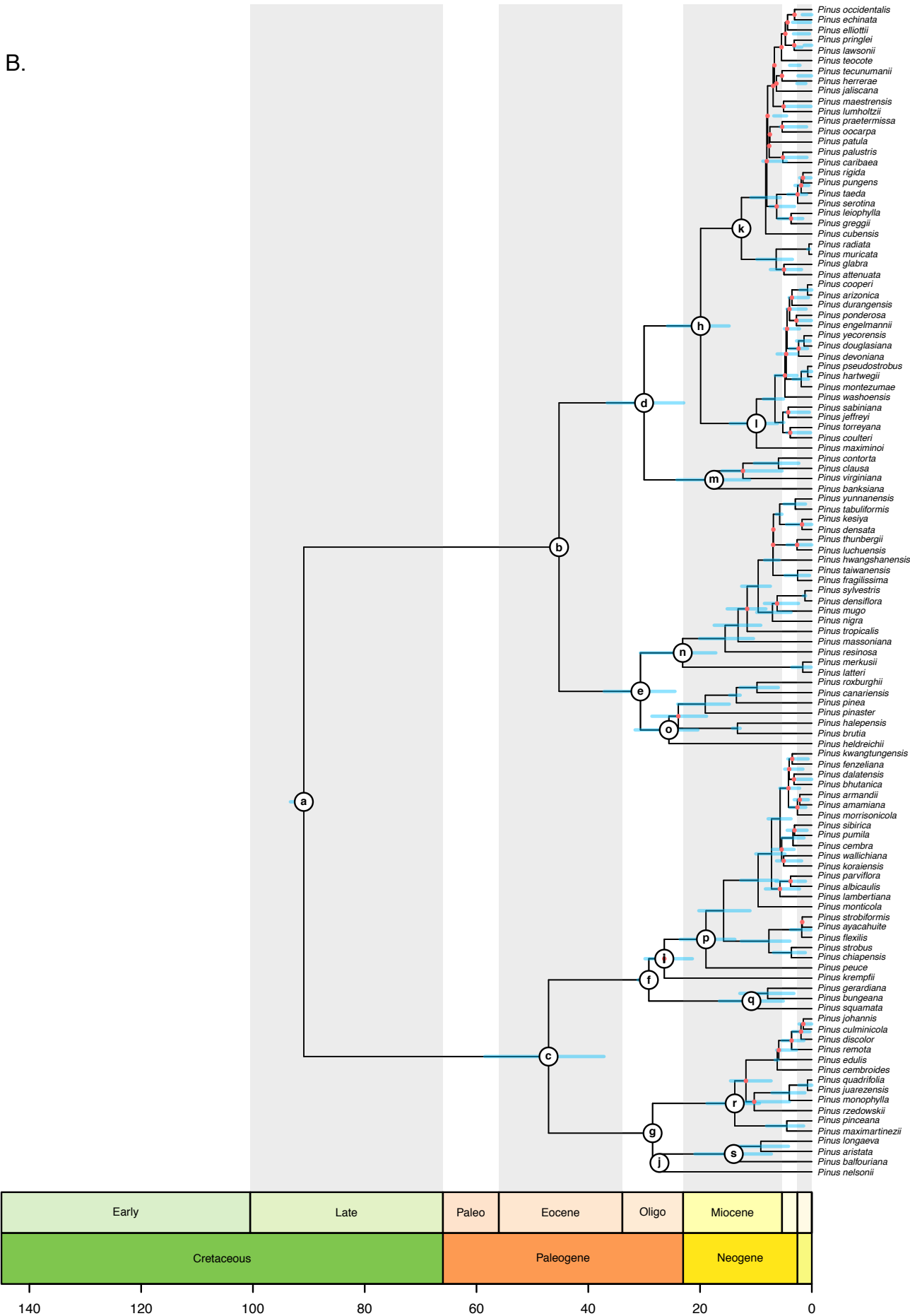

C.

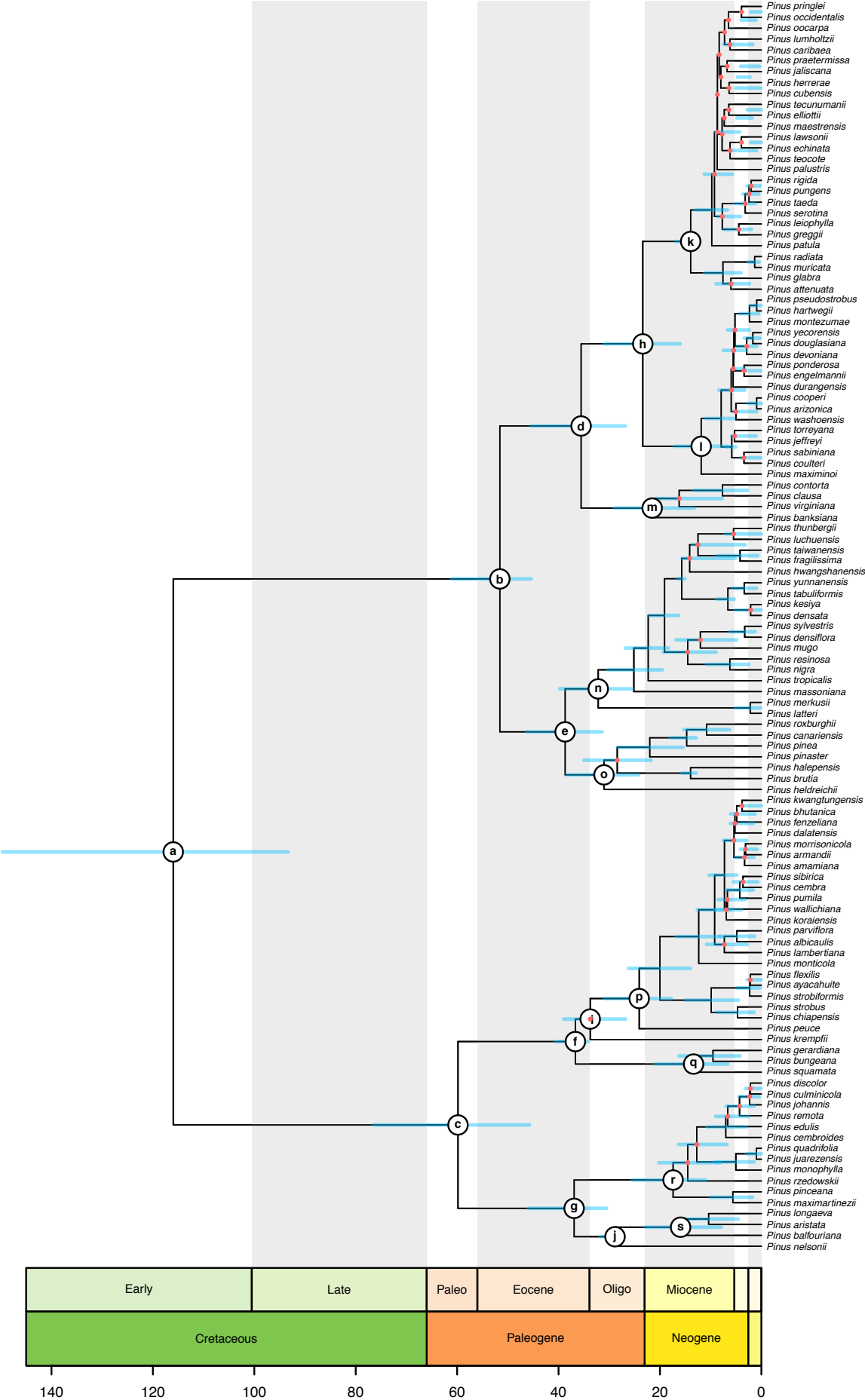

D.

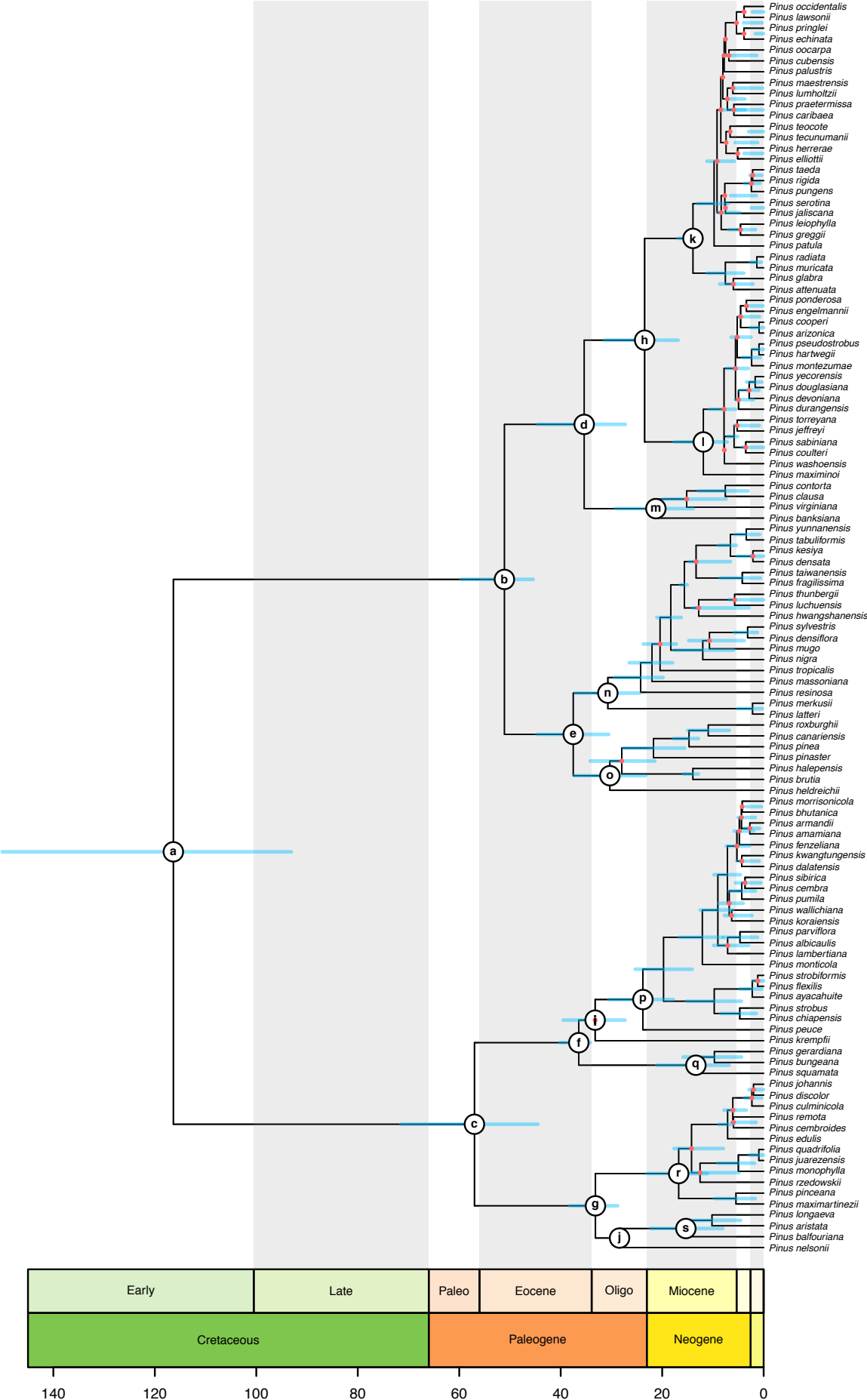

E.

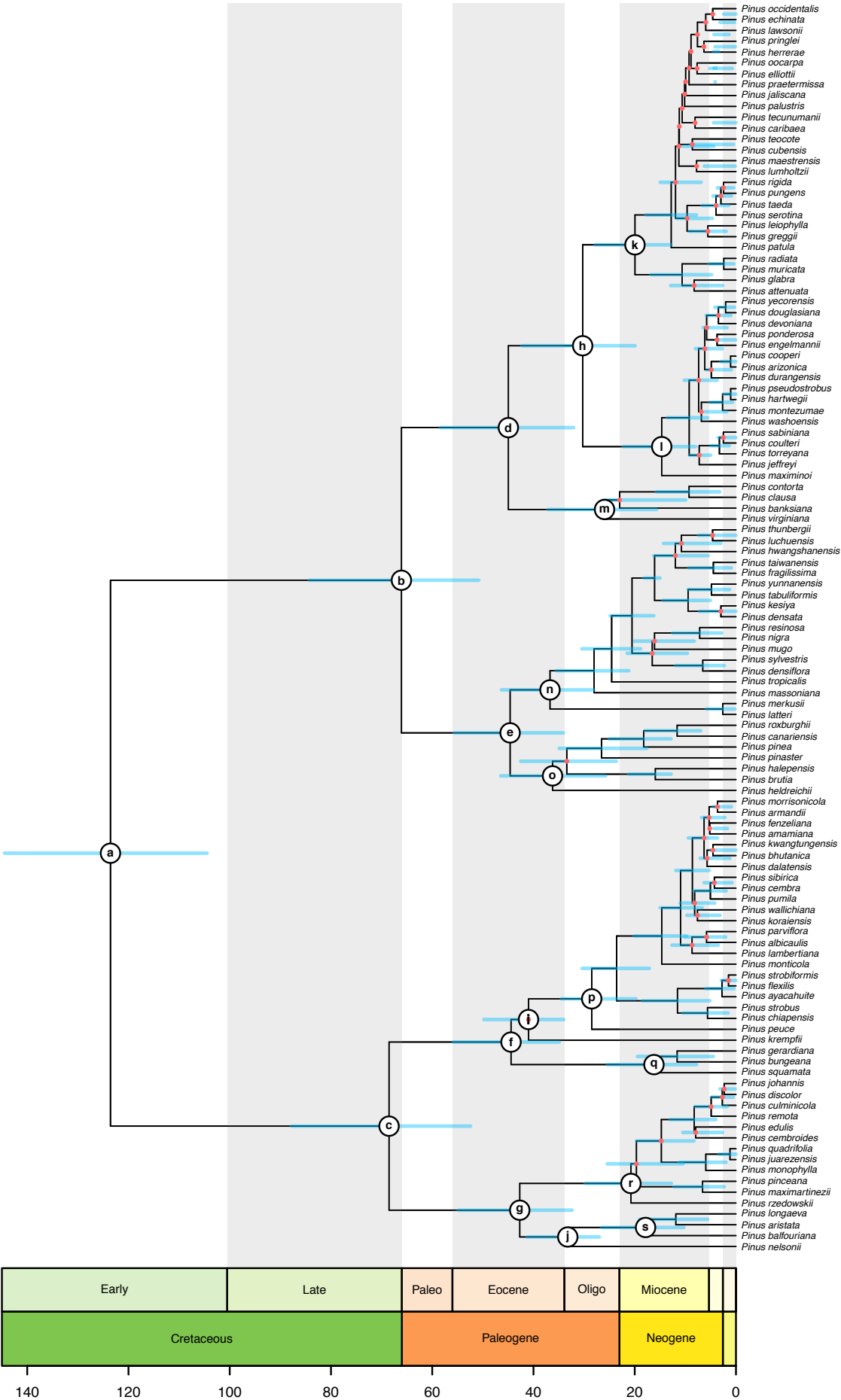

Supplement: Supplementary file 1 — Additional maximum clade credibility (MCC) trees. The trees originate from the node dating (ND) method with narrow prior calibration densities (NDn), with broad prior calibration densities (NDb), and from the fossilized birth-death (FBD) method. Each of these three methods was used in combination of either a small (s) or a large (l) fossil set. The following MCC trees are shown: (A) NDns, (B) NDnl, (C) NDbs, (D) NDbl, (E) FBDs. Note that the MCC tree for FBDl is given in Fig. 2. Nodes with red dots indicate Bayesian posterior probabilities lower than 0.95, while all other nodes have posterior probabilities higher than 0.95. Light blue lines on nodes represent the 95% highest posterior density (HPD) of the inferred phylogenetic trees. The node labels (a-s) indicate those nodes represented in Fig. 3. The geological timescale is in million years. (PDF 204 kb) [file 12862_2017_941_MOESM1_ESM.pdf]

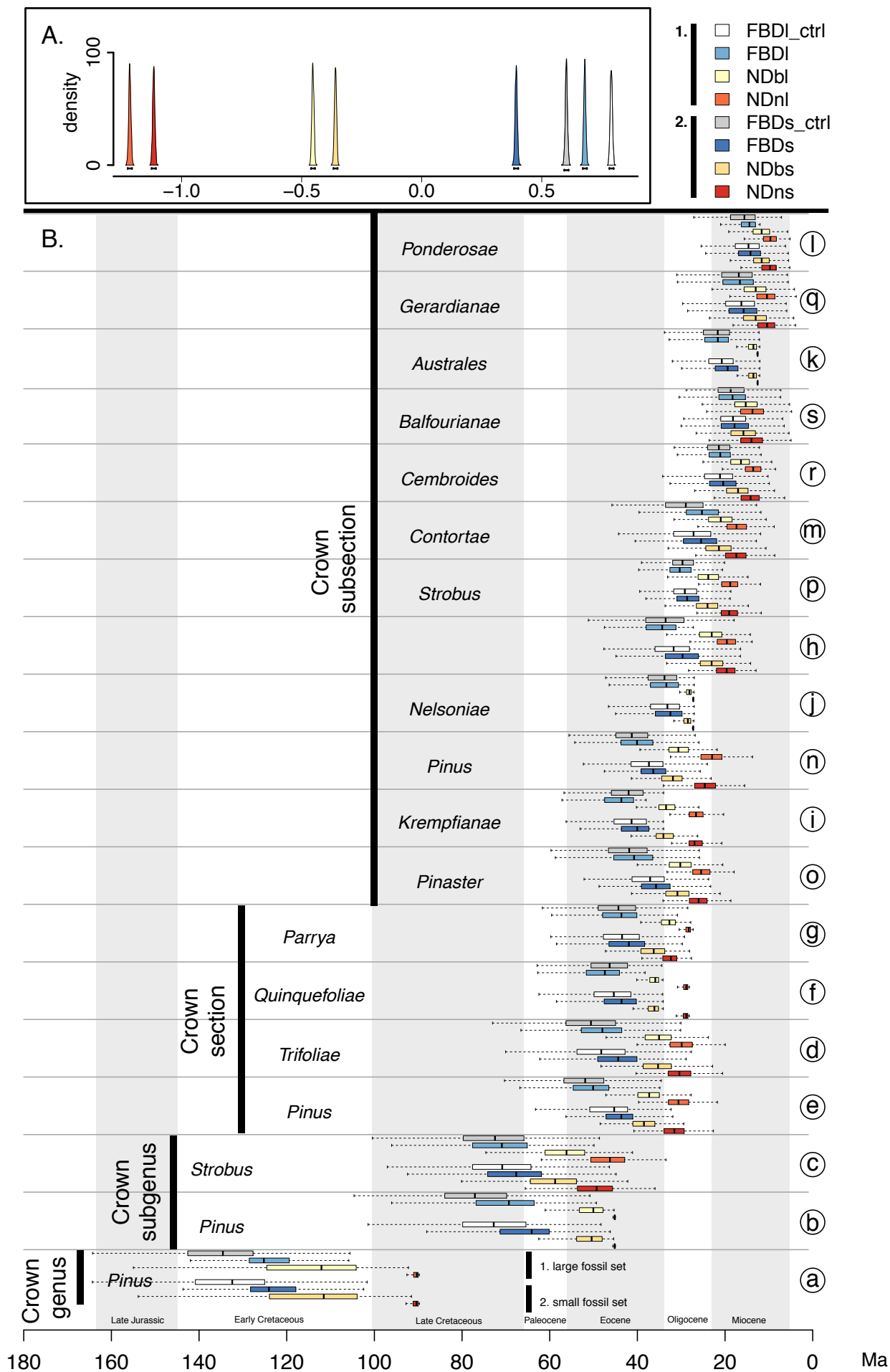

Supplement: Supplementary file 2 — Comparison of estimated node ages of the 19 major clades of Pinus across all applied dating approaches. A: Densities of effect sizes originate from a mixed-effect model and illustrate to what degree the estimated node ages differ among dating approaches (different colors; see legend) and among fossil sets (1. darker colors for the large, 2. brighter colors for the small fossil set; see legend). The 95% confidence intervals of effect sizes are illustrated with a line below the density curves. Non-overlap of these intervals indicates significant difference on node ages among all 19 nodes. B: Boxplots illustrate the estimated node ages across dating approaches and fossil sets for the major clades (a-s illustrated in Fig. 2). Whiskers span the 95% highest probability density (HPD), while boxes span the 50% HPD, with the median node age indicated by a vertical bar. The x-axis indicates the geological time in million years. The following abbreviations are used. FBD: fossilized birth-death method; ND: node dating method; l: analyses based on the large fossil set; s: analyses based on the small fossil set; n: narrow calibration priors in ND based on the geological age of the respective fossil; b: broad calibration priors in ND. (PDF 116 kb) [file 12862_2017_941_MOESM2_ESM.pdf]

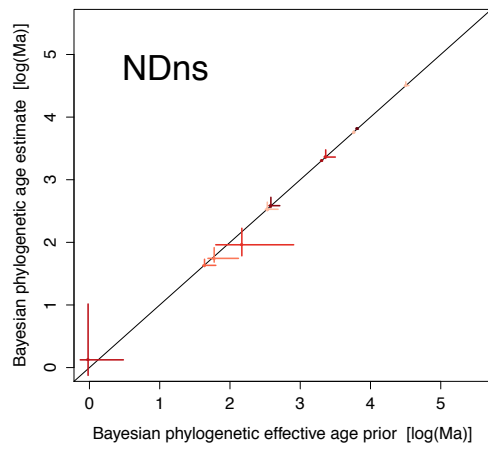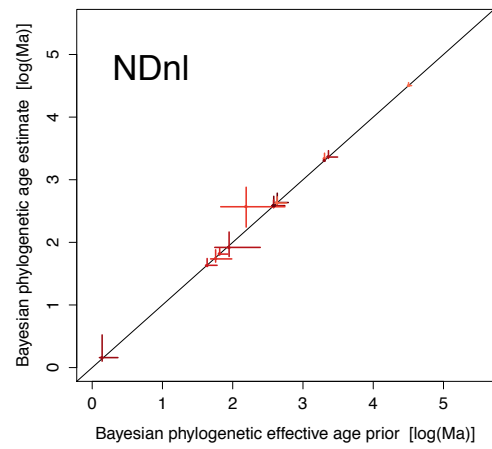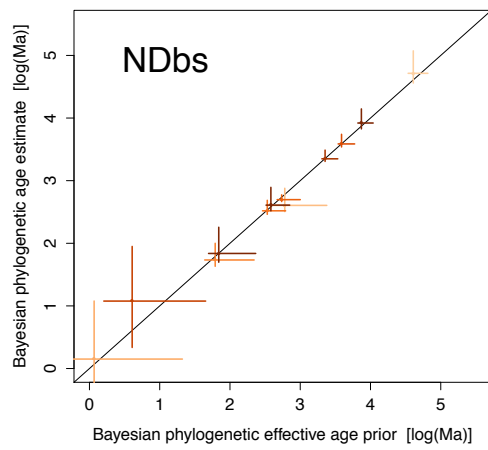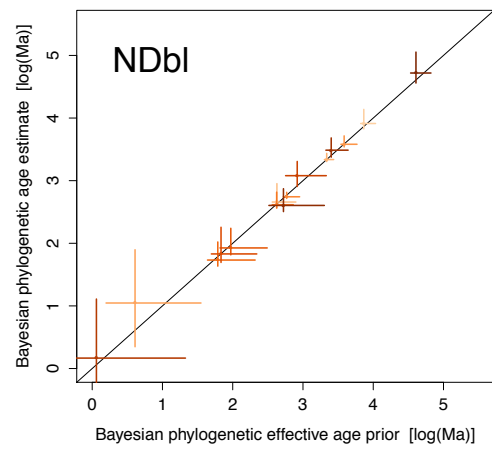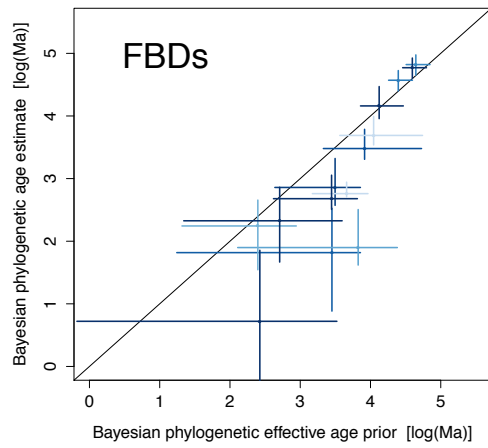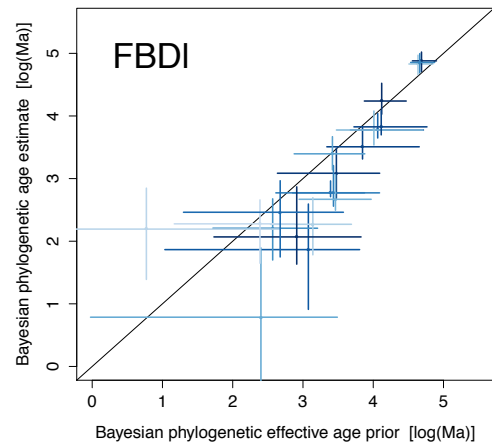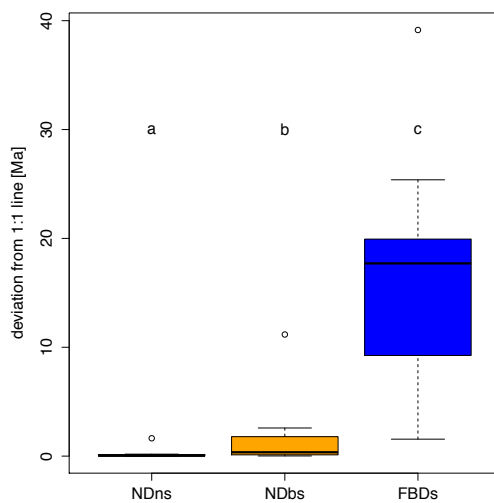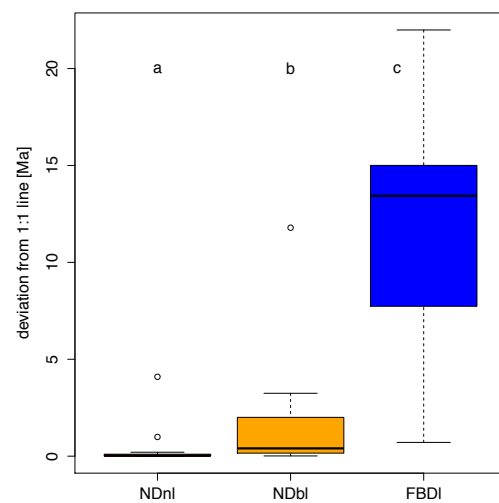

Supplement: Supplementary file 3 — Comparison of the effective age prior density against the posterior calibration densities (Bayesian phylogenetic age estimate) for the three dating approaches used (FBD: fossilized birth-death (blue); NDn/b: node dating with narrow (red) and broad (orange) prior distributions; s/l: small (A) and large (B) fossil sets. Boxplots represent the absolute deviation from the 1:1 line, while letters indicate significant differences in absolute deviations at the level of p = 0.05 (based on a paired Wilcox test). (PDF 116 kb) [file 12862_2017_941_MOESM3_ESM.pdf]

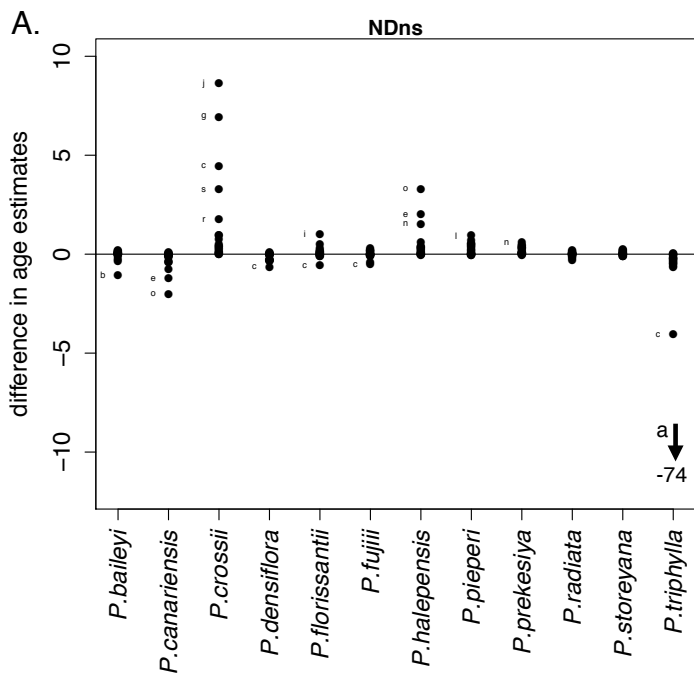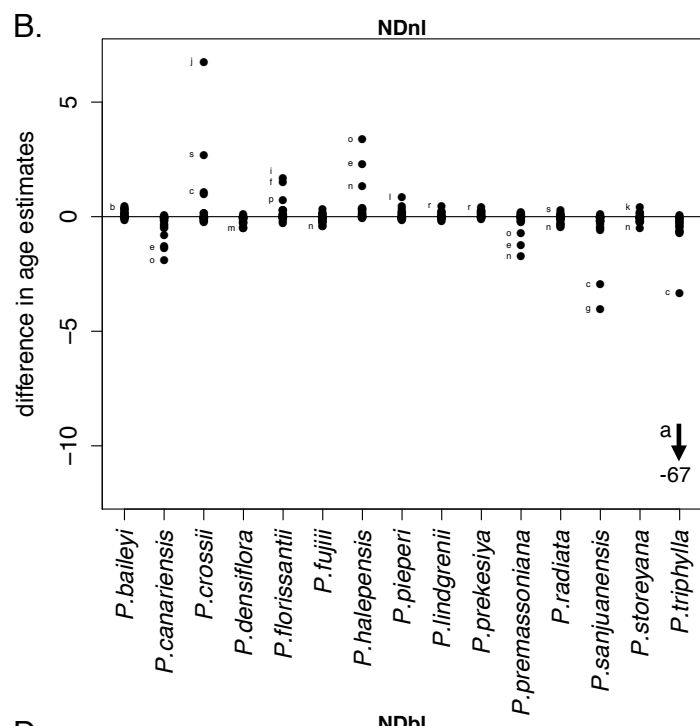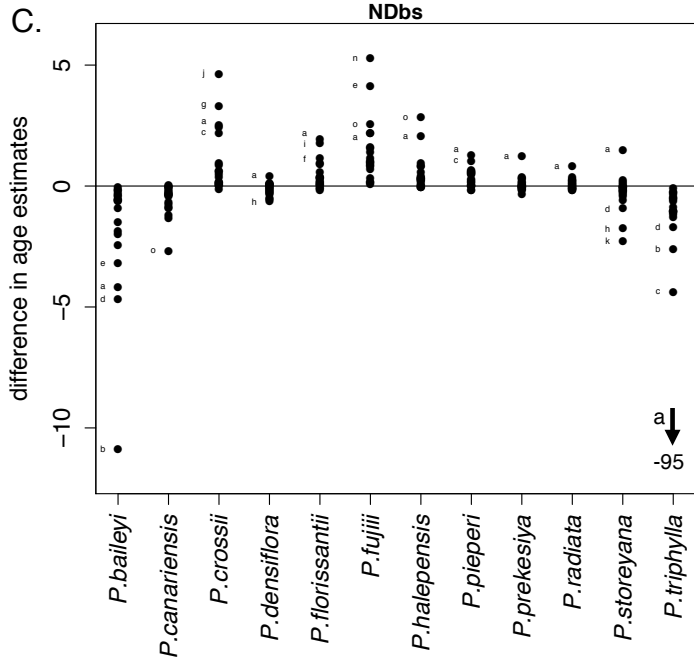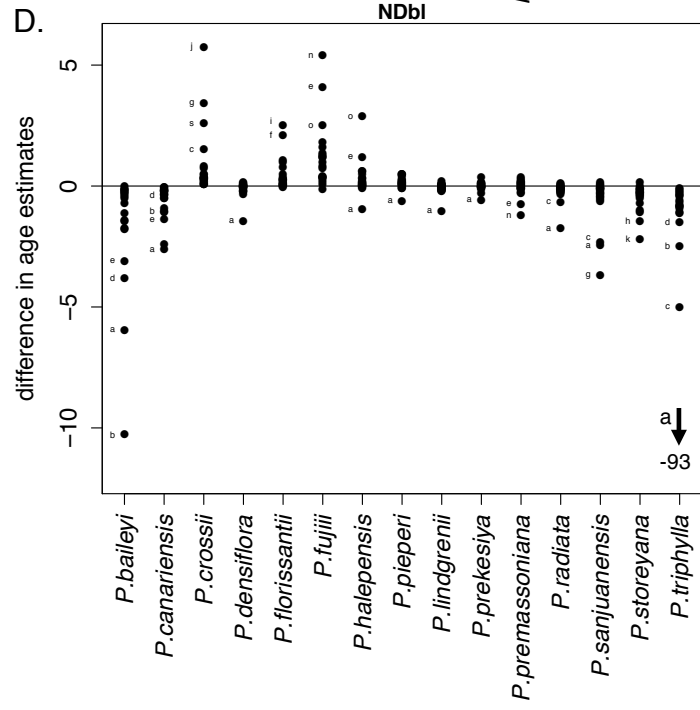

Supplement: Supplementary file 4 — Sensitivity of the time calibration to single fossil exclusion for the node dating approaches (ND). This test measures the difference in age estimates of the 19 major nodes (a-s, see also Fig. 2) when keeping versus removing single calibration constraints (fossil, labeled on x-axis) at a time. NDns and NDnl are based on narrow prior calibration densities using the small (A) and the large (B) fossil set, respectively. NDbs and NDbl are based on broad prior calibration densities using the small (C) and the large (D) fossil set, respectively. Letters (see Fig. 2 for assignment) indicate nodes with highest deviations. (PDF 125 kb) [file 12862_2017_941_MOESM4_ESM.pdf]

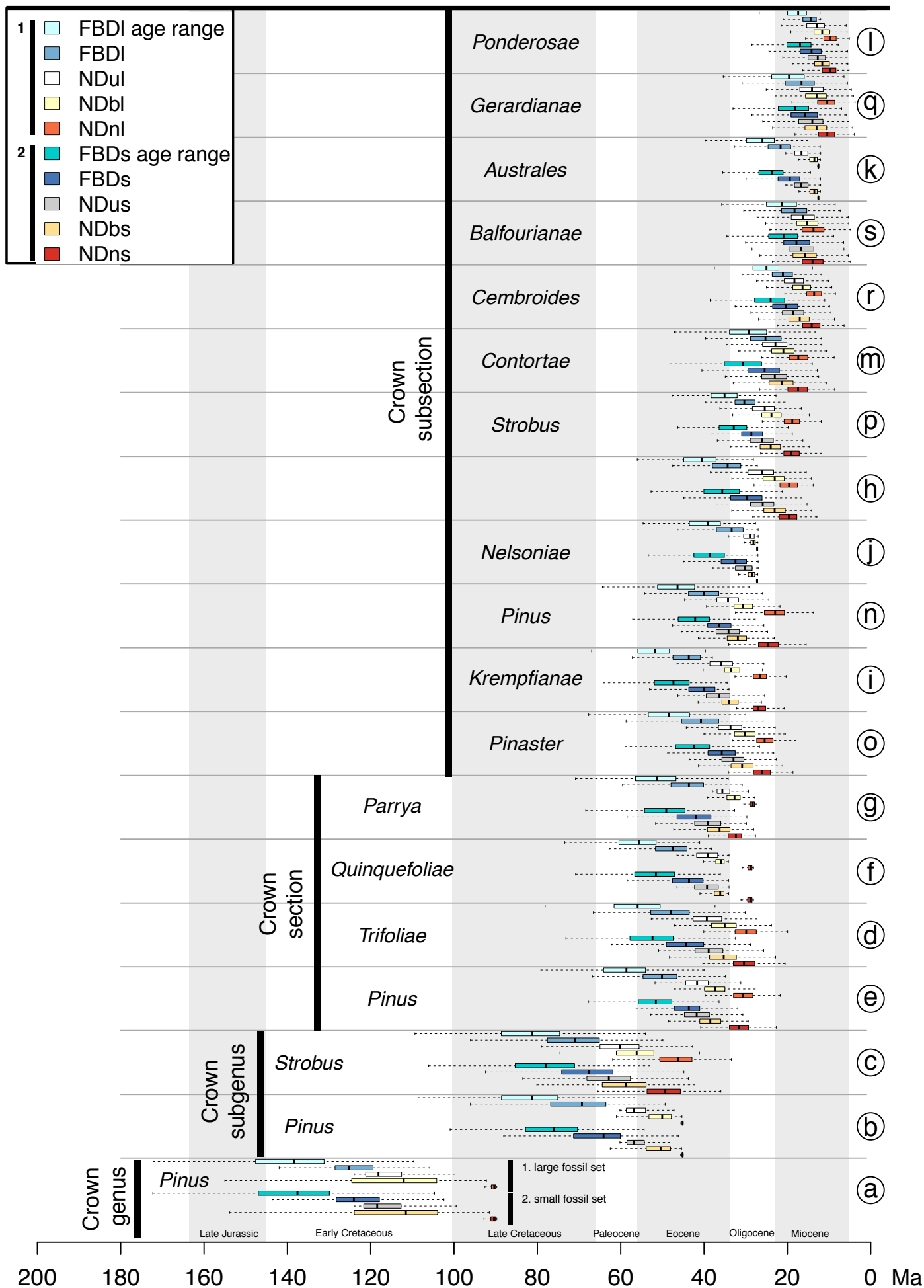

Supplement: Supplementary file 5 — Comparison of estimated node ages of the 19 major clades of Pinus across all applied dating approaches. Boxplots illustrate the estimated node ages across dating approaches and fossil sets for the major clades (a-s illustrated in Fig. 2 ). Whiskers span the 95% highest probability density (HPD), while boxes span the 50% HPD, with the median node age indicated by a vertical bar. The x-axis indicates the geological time in million years. The following abbreviations are used. FBD: fossilized birth-death method using “tip date” approach. FBD age range using “age range” approach. ND: node dating method; l: analyses based on the large fossil set; s: analyses based on the small fossil set; n: narrow log normal calibration priors in ND based on the geological age of the respective fossil; b: broad log normal calibration priors in ND; u: uniform calibration priors. The lower and upper limits of the uniform distribution represent the 2.5% and 97.5% CI levels used for the log-normal priors in the NDb method. (PDF 68 kb) [file 12862_2017_941_MOESM5_ESM.pdf]

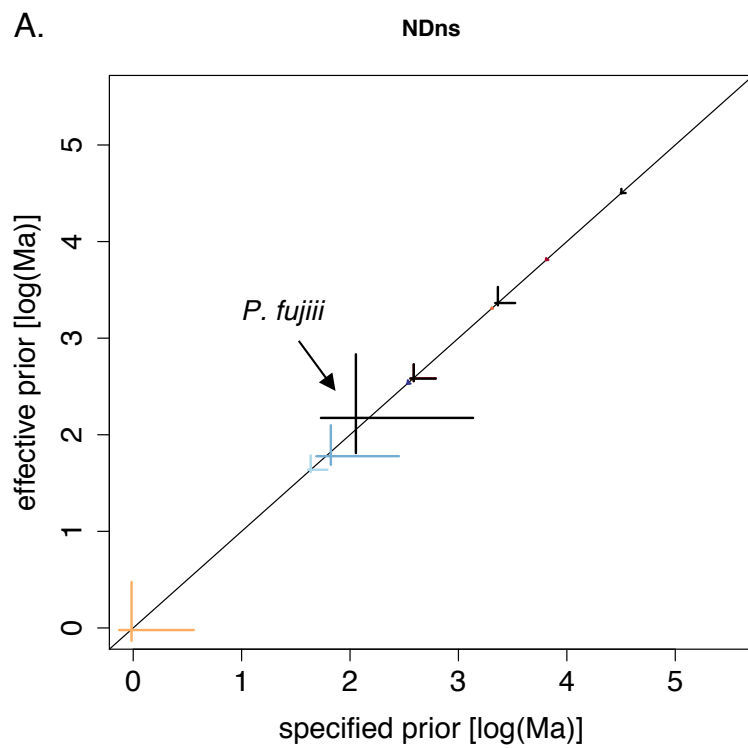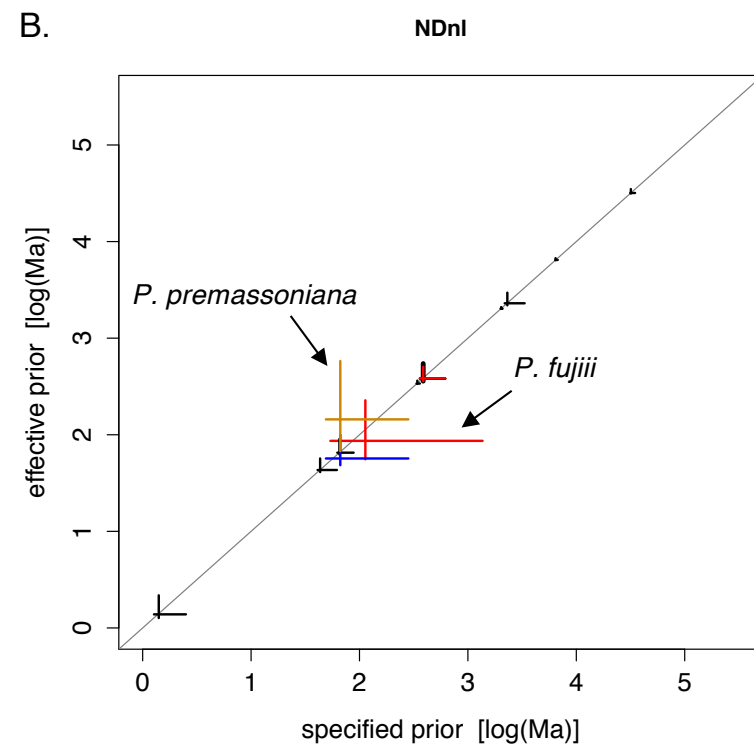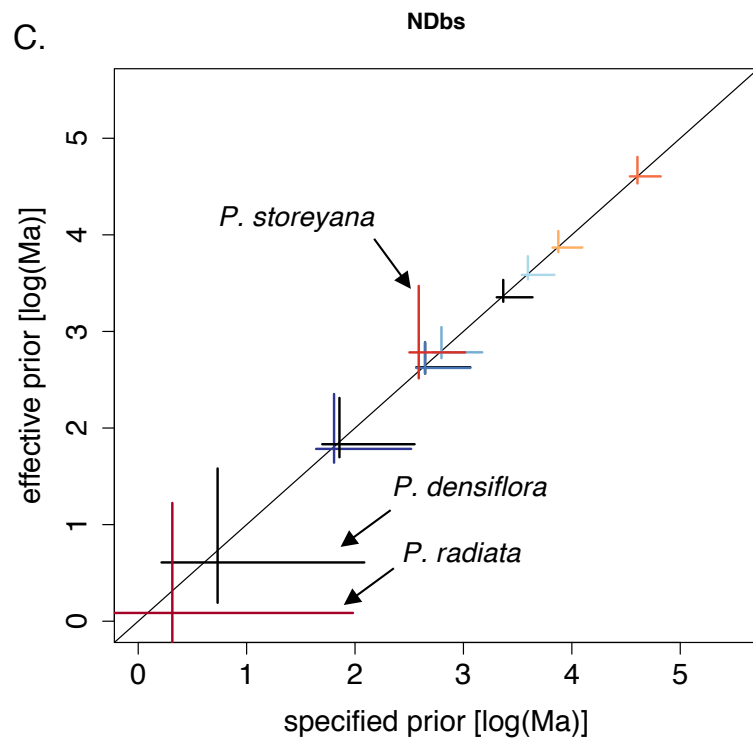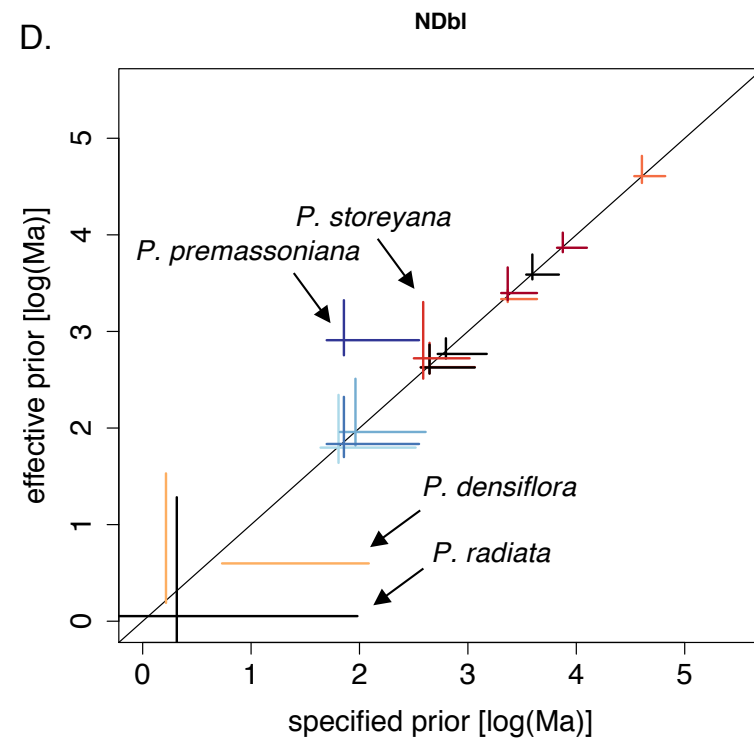

Supplement: Supplementary file 6 — Comparison of the specified calibration prior in BEAUti (log-transformed) against the effective calibration prior (log-transformed), as estimated without sequence data, and illustrated for all node dating approaches (ND). NDns and NDnl are based on narrow prior calibration densities using the small (A) and the large (B) fossil set, respectively. NDbs and NDbl are based on broad prior calibration densities using the small (C) and the large (D) fossil set, respectively. Labels are only given for fossil constraints with high deviance from the 1:1 line. (PDF 69 kb) [file 12862_2017_941_MOESM6_ESM.pdf]
