# Supplementary material for: Fossils matter: improved estimates of divergence times in Pinus reveal older diversification
Source: BMC Evol Biol. 2017 Apr 4;17:95. doi: 10.1186/s12862-017-0941-z (PMC5381128; doi:10.1186/s12862-017-0941-z)
Supplement: Supplementary file 7 — Overview of fossils used in this study. The following abbreviations and symbols are used: FBD: fossilized birth-death method; ND: node dating method; l: denotes analyses based on the large fossil set; s: denotes analyses based on the small fossil set; n: narrow calibration priors in ND based on the geological age of the respective fossil; b: broad calibration priors in ND. “x” in tables indicates which fossil was used in the different fossil sets. Asterisks on fossil numbers represent those used in the large fossil set only. A: the fossils used for calibration constraints are listed with the minimum age of each fossil listed. Table A1: Summary of fossils used in each fossil set and the geological layer (and the time scale thereof in Ma) in which the fossils were excavated with the corresponding references. Table A2: Specified prior calibration densities used for Bayesian clock methods. Fig. A1: Illustration of taxonomic assignment for each fossil in ND. Fig. A2: Illustration of taxonomic assignments for each fossil in FBD. Red dots indicate fossils that were allowed to be placed anywhere within the corresponding clade. Black dots illustrate fossils that were allowed to be placed anywhere along the indicated branch. B: Descriptions of alternative hypotheses (AH). Table A3: Summary of fossils used in each fossil set of the alternative hypotheses (AH). Fig. A3: Illustration of taxonomic assignment of fossils in the four alternative hypotheses. Fig. A4: Estimated ages by each of the four alternative hypotheses compared to the primary age estimates in Fig. 3. Boxplots illustrate the estimated node ages across dating approaches and fossil sets for the major clades (a-s illustrated in Fig. 2). Whiskers span the 95% highest probability density (HPD), while boxes span the 50% HPD, with the median node age indicated by a vertical bar. The x-axis indicates the geological time in million years. C: Fossil descriptions and justifications ordered by taxonomic groups. Bold-italic f [file 12862_2017_941_MOESM7_ESM.pdf]

Discussion of fossils used in this study

Pinaceae have a rich fossil record, but most specimens do not easily lend themselves to phylogenetic analysis. For example, Pinaceae fossils are usually found as isolated seeds, seed cones, or needles, and therefore whole-plant reconstructions recording complete morphological and anatomical information for fossil taxa are rare (e.g., *Pinus arnoldii* Miller; Klymiuk *et al.* 2011) . It has therefore been difficult to resolve phylogenetic relationships among fossil Pinaceae, particularly among early members (see Smith & Stockey, 2001) .

Given these difficulties, we do not attempt to resolve the phylogenetic placement of particular fossils using an explicit analysis. Instead, we identify likely synapomorphies based on the distribution of derived traits among extant species and then use these traits to link fossils with particular clades. Because this approach is fundamentally a hypothesis of how fossils are related to modern taxa, we employ several different fossil calibration schemes that serve as different hypotheses of fossil age constraints. Broadly speaking, we have divided calibration fossils into two different sets that differ in our degree of confidence concerning their phylogenetic placement. The first set of fossils (the 'short list') consists of taxa that exhibit traits we feel are clear synapomorphies for living clades, or of taxa that are morphologically indistinguishable from extant species. Such a conservative approach may bias analyses towards inferring young ages, however, because older and often more ambiguous fossil taxa are not considered. The second set (the 'long list') therefore uses a larger number of fossils and somewhat relaxes the criterion of unambiguous synapomorphies. In the 'long list' we often follow the taxonomic assignment of the original authors, where there is commonly a tendency to link fossils with extant subclades within *Pinus*. Analyses of these two fossil sets can be directly compared for consistency, and we believe they provide a reasonable bracket on ages within *Pinus*. Nevertheless, it is inevitable that some fossils from either set are not as easy to interpret as others, either because they are not as well known as other fossil taxa or because that the synapomorphies they possess could be interpreted as homoplastic. We have therefore developed four additional hypotheses based on either eliminating particular problematic fossils or reinterpreting their likely phylogenetic position.

In part A, we summarize the fossils we used for the "short" and "long" lists, as well as their minimum ages and their phylogenetic placement. These are the fossil analyses that are primarily discussed in the main text. In part B, we summarize four alternative hypotheses (AH) using a subset of these fossils. In part C, we provide more detailed descriptions and justifications for each fossil used in the study. Throughout this description, our taxonomy follows the sectional and subsectional classification of Gernandt *et al*. (2005).

A. Primary fossil analyses

<u>Small fossil set: the "short list"</u>

- *Pinus baileyi.* 45 Ma; stem section *Pinus*
- *Pinus canariensis.* 12.8 Ma; stem (*P. canariensis + P. roxburghii)* clade
- *Pinus crossii.* 27 Ma; stem subsection *Balfourianae*
- *Pinus densiflora.* 1.1 Ma; stem *P. densiflora*
- *Pinus florissantii.* 34 Ma; stem subsection *Strobus*
- *Pinus fujiii.* 15 Ma; stem MRCA of *P. kesiya* + *P. tabuliformis*
- *Pinus haboroensis.* 65 Ma; stem subgenus *Pinus*
- *Pinus halepensis.* 12.8 Ma*;* stem *P. halepensis*
- *Pinus pieperi.* 5 Ma; (*P. coulteri* + *P. sabiniana)* clade divergence
- *Pinus prekesiya.* 5.3 Ma; *P. yunnanensis* – *P. kesiya* divergence
- *Pinus radiata.* 0.4 Ma; stem *P. radiata*
- *Pinus storeyana*. 12 Ma; member *Attenuatae* clade
- *Pinus triphylla*. 90 Ma; stem subgenus *Pinus*
- *Pinus yorkshirensis*. 129 Ma; stem *Pinus*

<u>Additional fossils used in the large fossil set: the "long list"</u>

- *Pinus delmarensis.* 38 Ma; stem subsection *Strobus*
- *Pinus lindgrenii.* 6 Ma; MRCA of *P. edulis* - *P. johannis* clade
- *Pinus premassoniana*. 5.3 Ma; stem *P. massoniana*
- *Pinus riogrande.* 27.2 Ma; member *Ponderosae* clade
- *Pinus sanjuanensis.* 27 Ma; stem subsection *Cembroides*
- *Pinus truckeensis.* 12 Ma; subsection *Ponderosae* within *P. ponderosa* - clade
- *Pinus weasmaii.* 3 Ma; stem *P. contorta*

Table A1. Overview of fossils used in the ‘short’ and ‘long’ list

	Fossils	NDns	NDnl	NDbs	NDbl	FBDs	FBDs_ctrl	FBDl	FBDl_ctrl	Geological timescale	References
1	*P. baileyi*	x	x	x	x	x	x	x	x	Thunder Mountain, Florida: **46 - 45 Ma**	(Erwin & Schorn, 2006)
2	*P. canariensis*	x	x	x	x	x	x	x	x	Styrian Basin: **16.3 - 12.8 Ma**	(Klaus, 1989)
3	*P. crossii*	x	x	x	x	x	x	x	x	*Creed Flora, Colorado: **27.2 Ma**	(Wolfe & Schorn, 1990)
*4	*P. delmarensis*							x		Del Mar Formation, California: **47.8 - 38 Ma**	(Axelrod, 1986)
5	*P. densiflora*	x	x	x	x	x	x	x	x	Lower part of Osaka Group in Pleistocene: **0.86 - 1.75 Ma**	(reviewed in Yamada *et al.*, 2014)
6	*P. florissantii*	x	x	x	x	x	x	x	x	Early Oligocene: **33.9 - 28.1 Ma**	(Axelrod, 1986)
7	*P. fujiii*	x	x	x	x	x	x	x	x	Lower to Upper Miocene: **23 - 5.3 Ma**	(reviewed in Yamada *et al.*, 2014)
8	*P. haboroensis*					x		x		Santonian - Maastrichtian: **85 - 65 Ma**	(Stockey & Nishida, 1986)
9	*P. halepensis*	x	x	x	x	x	x	x	x	Badenian: **16.3 - 12.8 Ma**	(Klaus, 1989)
10	*P. pieperi*	x	x	x	x	x	x	x	x	Mount Eden Flora: **6 - 5 Ma**	(Axelrod, 1986)
*11	*P. lindgrenii*		x		x			x		Chalk Hills Formation, Idaho: **7 - 6 Ma**	(Knowlton, 1901; Axelrod, 1986)
12	*P. prekesiya*	x	x	x	x	x	x	x	x	Xiaolongtain Formation: **11.6 - 5.3 Ma**	(Xing *et al.*, 2010)
*13	*P. premassoniana*		x		x			x		Shengxian Formation: **11.6 - 5.3 Ma**	(Ding *et al.*, 2013)
14	*P. radiata*	x	x	x	x	x	x	x	x	Santa Barbara Formation: **0.8 - 0.4 Ma**	(Axelrod, 1986)
*15	*P. riogrande*							x		Creed Flora, Colorado: **27.2 Ma**	(Axelrod, 1986)
*16	*P. sanjuanenesis*		x		x			x		*Creed Flora, Colorado: **27.2 Ma**	(Axelrod, 1986)
17	*P. storeyana*	x	x	x	x	x	x	x	x	*Coal Valley Formation: **12.5 Ma**	(Axelrod & Cota, 1993)
18	*P. triphylla*	x	x	x	x	x	x	x	x	Turonian: **93.9 - 89.7 Ma**	(Robison, 1977)
*19	*P. truckeensis*							x		Coal Valley Formation, Celetom Quarry, Late Miocene: **12 Ma**	(Axelrod, 1986)
*20	*P. weasmaii*							x		Glenns Ferry Formation, Idaho, Pliocene, **5.3 - 2.58 Ma**	(Miller, 1992)
21	*P. yorkshirensis*					x		x		Wealden Formation: **129 - 131 Ma**	(Ryberg *et al.*, 2012)

Table A2. Specified prior calibration densities used for Bayesian clock methods

Fossil		NDn				NDb				FBD	FBD
		offset	2.5%	median	97.5%CI	offset	2.5%	median	97.5%CI	tip date	age range
1	*P. baileyi*	45	45	45.1	46	45	45.7	48.2	60.2	45	[45, 60.2]
2	*P. canariensis*	12.8	12.9	13.3	16.3	12.8	13.0	14.1	21.4	12.8	[12.8, 21.4]
3	*P. crossii*	27.2	27.2	27.3	27.6	27	27.3	29.0	38.0	27	[27, 38]
*4	*P. delmarensis*	-	-	-	-	-	-	-	-	38	[38, 51.4]
5	*P. densiflora*	0.86	0.878	0.985	1.75	1.1	1.24	2.08	8.05	1.1	[1.1, 8.05]
6	*P. florissantii*	28.1	28.2	28.9	33.9	34	34.4	36.4	46.5	34	[34, 46.5]
7	*P. fujiii*	5.3	5.65	7.80	23.0	15	15.2	16.4	23.9	15	[15, 23.9]
8	*P. haboroensis*	-	-	-	-	-	-	-	-	65	[65, 86.8]
9	*P. halepensis*	12.8	12.9	13.3	16.3	12.8	13.0	14.1	21.4	12.8	[12.8, 21.4]
10	*P. pieperi*	5	5.02	5.14	6.00	5	5.16	6.09	12.4	5	[5, 12.4]
*11	*P. lindgrenii*	6	6.02	6.14	7	6	6.17	7.12	13.6	6	[6, 13.6]
12	*P. prekesiya*	5.3	5.42	6.19	11.6	5.3	5.46	6.40	12.8	5.3	[5.3, 12.8]
*13	*P. premassoniana*	5.3	5.42	6.19	11.6	5.3	5.46	6.4	12.8	5.3	[5.3, 12.8]
14	*P. radiata*	0.4	0.408	0.457	0.803	0.4	0.536	1.37	7.25	0.4	[0.4, 7.25]
*15	*P. riogrande*	-	-	-	-	-	-	-	-	27.2	[27.2, 38.3]
*16	*P. sanjuanenesis*	27.2	27.2	27.3	27.6	27	27.3	29	38	27	[27, 38]
17	*P. storeyana*	12.5	12.5	12.6	12.9	12	12.2	13.3	20.4	12	[12, 20.4]
18	*P. triphylla*	89.7	89.8	90.3	93.9	90	93.2	100	124	90	[90, 124]
*19	*P. truckeensis*	-	-	-	-	-	-	-	-	12	[12, 20.4]
*20	*P. weasmaii*	-	-	-	-	-	-	-	-	3	[3, 10.2]
21	*P. yorkshirensis*	-	-	-	-	-	-	-	-	129	[129, 160]

Figure A1. Fossil placement for primary node dating analyses

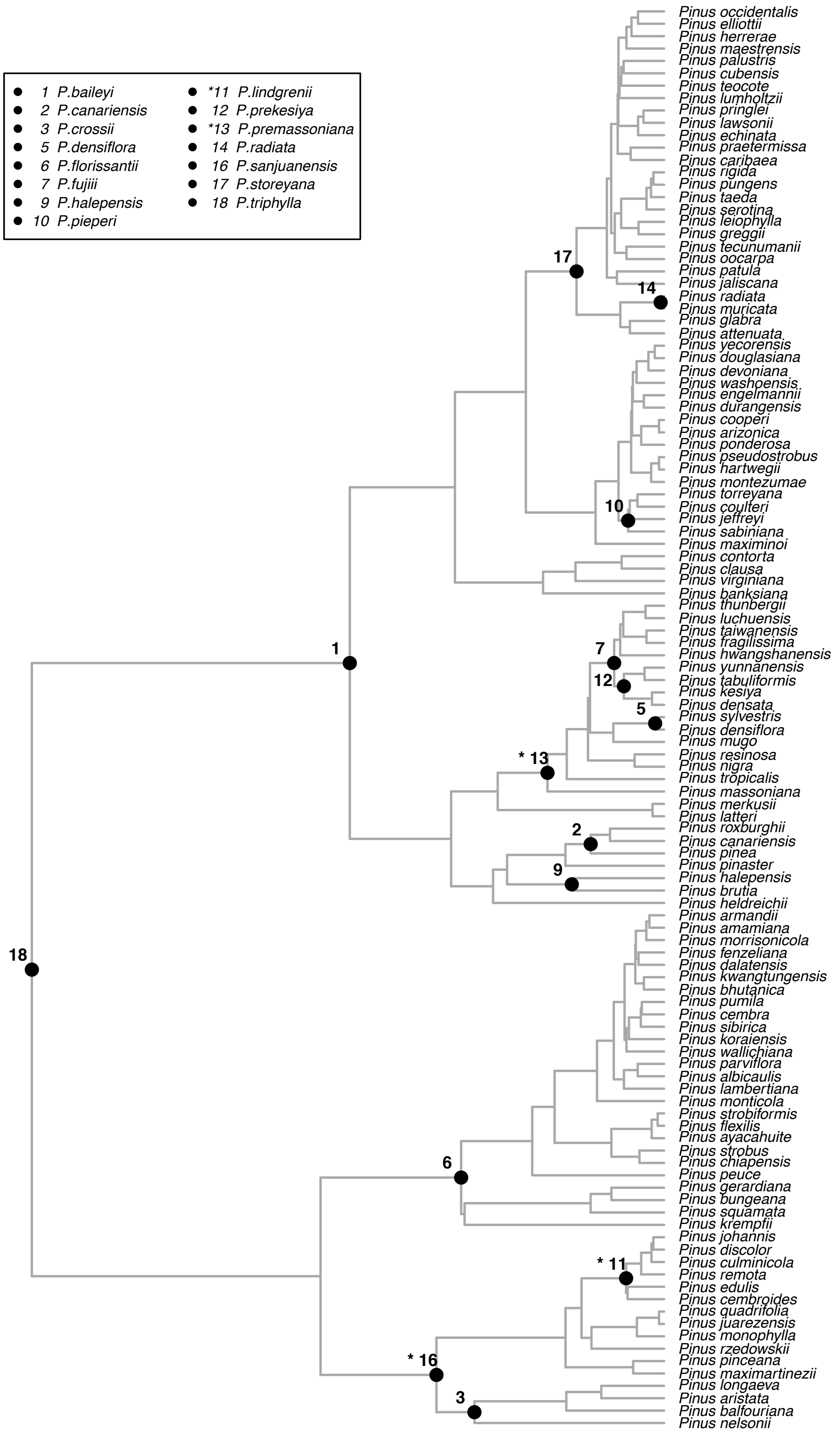

Figure A2. Fossil placement for primary fossilized birth-death analyses

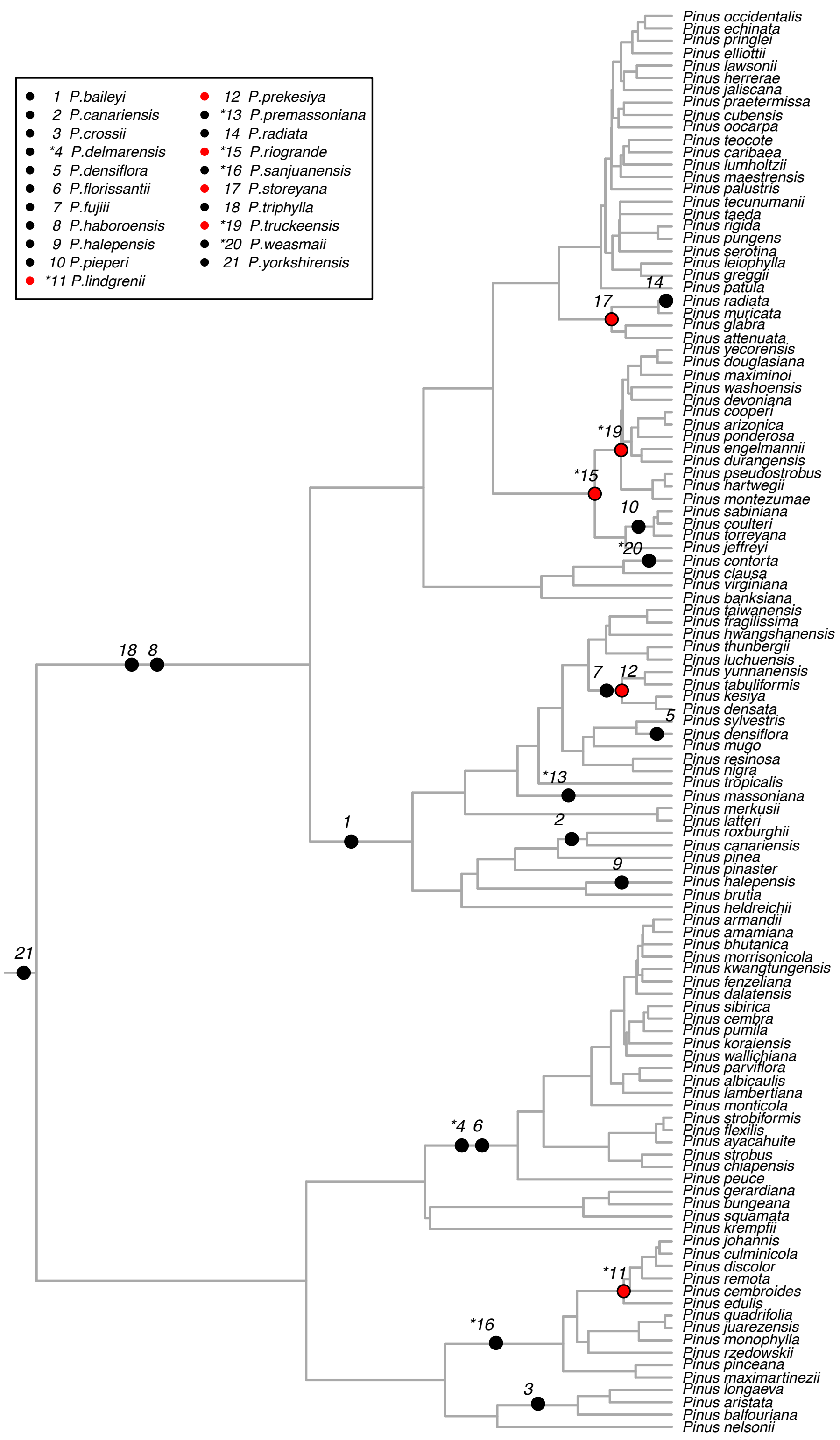

B. Additional hypotheses

Below we introduce four additional hypotheses based on different interpretations of fossils or the exclusion of potentially uncertain fossil taxa. The rationale behind these additional analyses was to test if the results presented in the main paper are robust and not simply an effect of a specific hypothesis regarding fossil choice and placement. In particular, published descriptions of pine fossil taxa often classify them into infrageneric subclades (sections, subsections, etc.) based on general morphological similarity rather than robust synapomorphies. As our primary analyses (the 'short' and 'long' lists) sometimes relied on these original assessments, we assess the uncertainty from these infrageneric assignments in these additional runs by removing potentially uncertain taxa. Here we present four alternative hypotheses that we feel test the effect of including or excluding the most uncertain taxa.

Alternative Hypothesis 1 (AH1)

This fossil set is a modified version of the 'short list'. It represents the most conservative application of our fossil list by removing all fossils whose placement is potentially doubtful due to either the lack of clear synapomorphies or uncertainties in how to interpret them. The removed fossil taxa include: *P. fujiii, P. haboroensis, P. pieperi, P. prekesiya,* and *P. triphylla*.

Alternative Hypothesis 2 (AH2)

This is the same as AH1, but we included *P. yorkshirensis* to date the oldest node in the tree. In order to do this, we included an outgroup species in ND (*Picea sitchensis*), although for FBD, no changes were needed.

Alternative Hypothesis 3 (AH3)

This fossil set is based on AH1, but in this case we retained *P. triphylla* and *P. haboroensis* to test the effect of including these uncertain taxa in estimating the split between Subgenus *Strobus* and Subgenus *Pinus*.

Alternative Hypothesis 4 (AH4)

This fossil set is based on the original 'long list' but we removed several uncertain fossils. These changes are more likely to affect the FBD analyses than the ND analyses, as many of the removed fossils were not included in the ND analyses. The removed fossil taxa include: *P. delmarensis, P. premassoniana, P. riogrande, P. sanjuanensis* and *P. truckeensis.*

Table B1. Summary of fossils used in the alternative hypotheses

	Fossils	AH1	AH2	AH3	AH4
1	*P. baileyi*	x	x	x	x
2	*P. canariensis*	x	x	x	x
3	*P. crossii*	x	x	x	x
4	*P. delmarensis*				
5	*P. densiflora*	x	x	x	x
6	*P. florissantii*	x	x	x	x
7	*P. fujiii*				x
8	*P. haboroensis*			x	x
9	*P. halepensis*	x	x	x	x
10	*P. pieperi*				x
11	*P. lindgrenii*	x	x	x	x
12	*P. prekesiya*				x
13	*P. premassoniana*				
14	*P. radiata*	x	x	x	x
15	*P. riogrande*				
16	*P. sanjuanenesis*				
17	*P. storeyana*	x	x	x	x
18	*P. triphylla*			x	x
19	*P. truckeensis*				
20	*P. weasmaii*				x
21	*P. yorkshirensis*		x	x	x

Figure B1. Fossil placement in alternative hypotheses

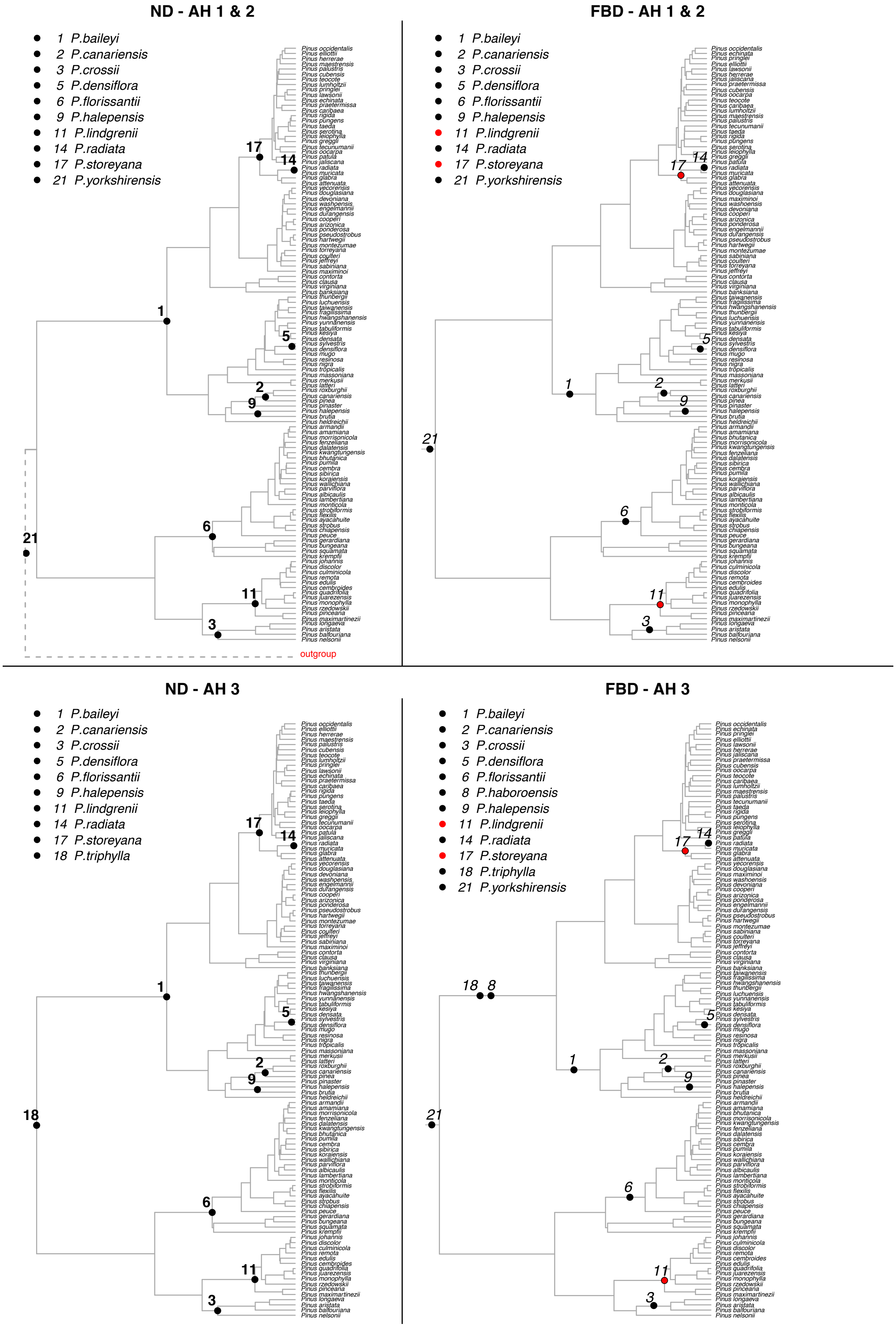

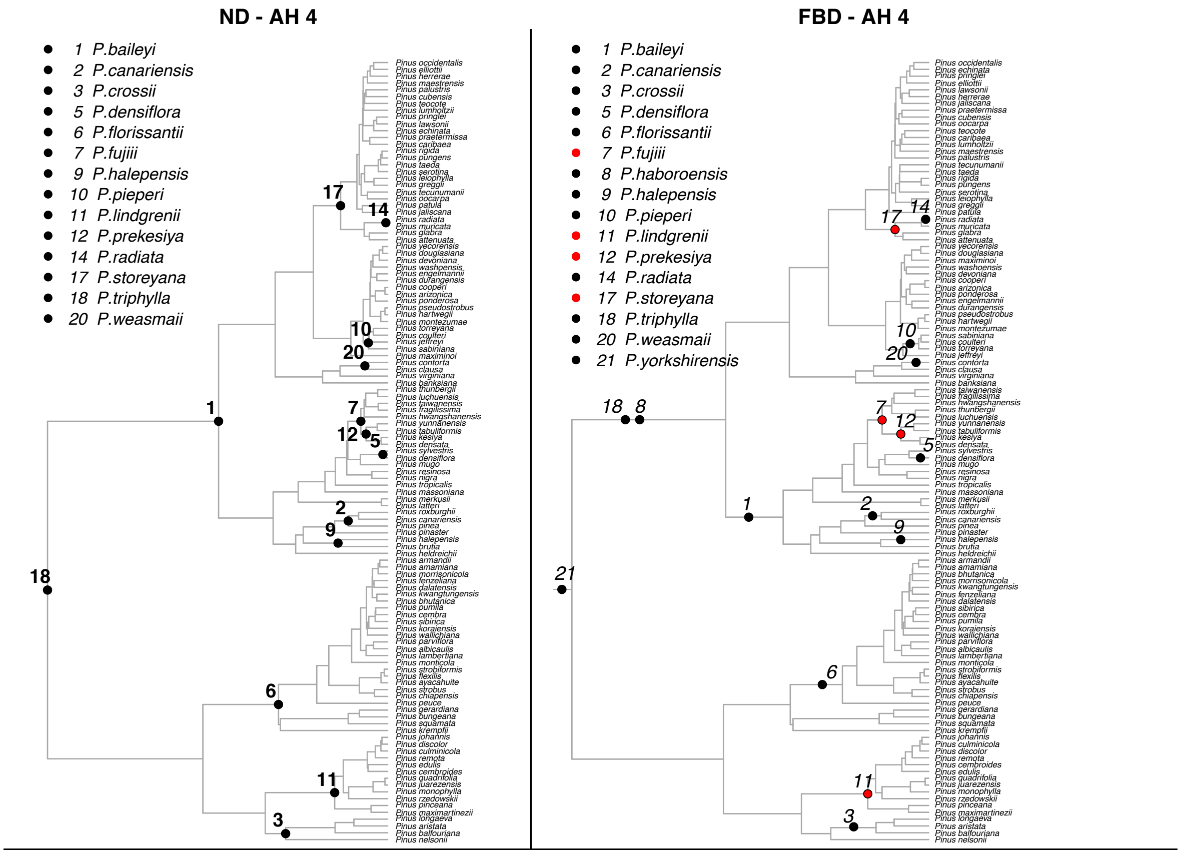
ND - AH 4
1 P.baileyi
2 P.canariensis
3 P.crossii
5 P.densiflora
6 P.florissantii
7 P.fujiii
9 P.halepensis
10 P.pieperi
11 P.lindgrenii
12 P.prekesiya
14 P.radiata
17 P.storeyana
18 P.triphylla
20 P.weasmaii
FBD - AH 4
1 P.baileyi
2 P.canariensis
3 P.crossii
5 P.densiflora
6 P.florissantii
7 P.fujiii
8 P.haboroensis
9 P.halepensis
10 P.pieperi
11 P.lindgrenii
12 P.prekesiya
14 P.radiata
17 P.storeyana
18 P.triphylla
20 P.weasmaii
21 P.yorkshirensis

Figure B2. Age estimates based on alternative fossil sets compared to standard sets

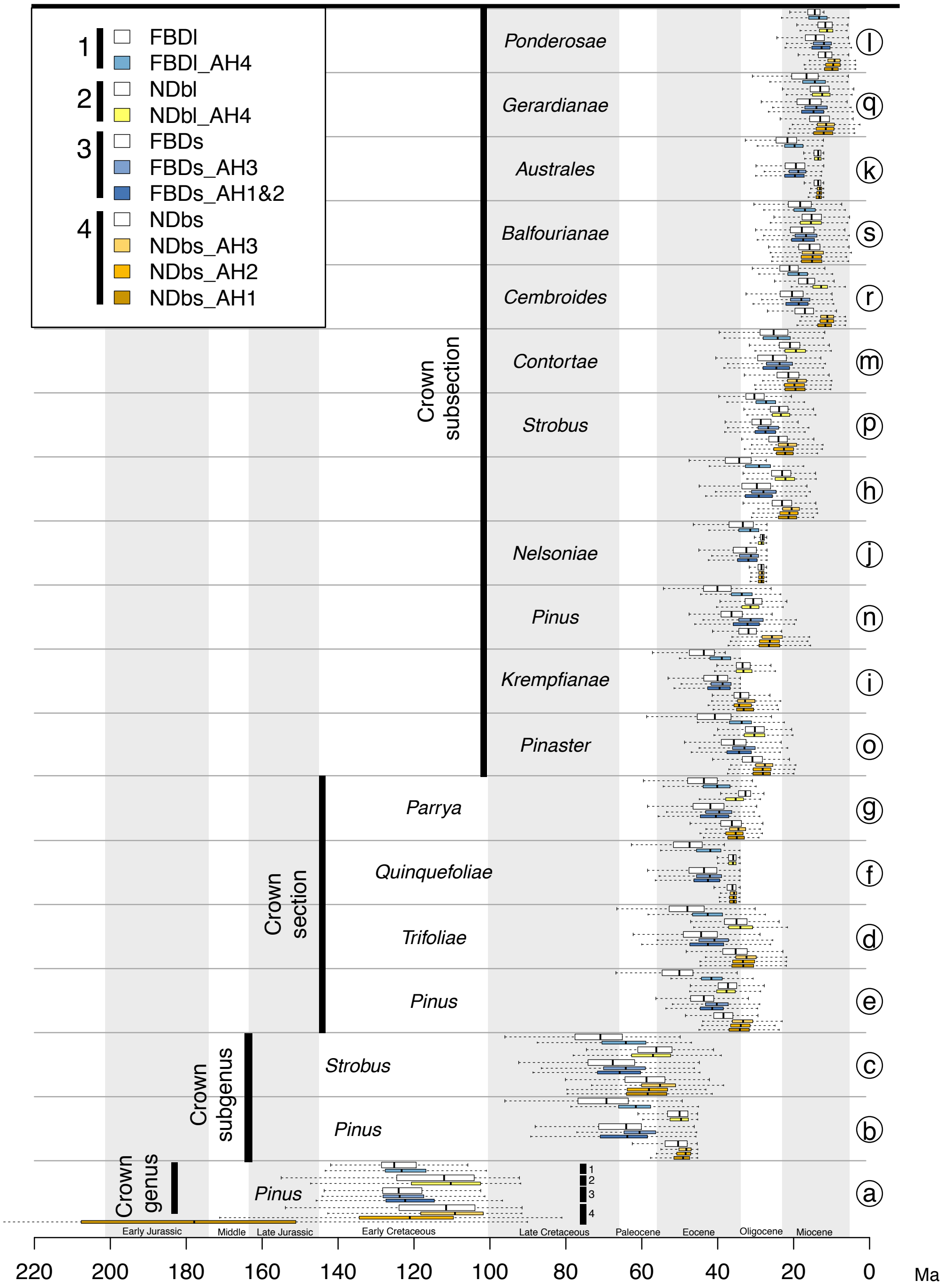

C. Fossil descriptions and justification

Genus *Pinus*

Pinus yorkshirensis Ryberg, Stockey, Hilton, Mapes, Riding et Rothwell

Location: Wealden Formation, England
Age: 131-129 Ma.

Discussion: These are the earliest well-dated cones that belong to the genus *Pinus* based on internal anatomy and external morphology. These features in particular include the presence of cone scales with apophyses and umbos, features unique to *Pinus* among extant Pinaceae (Ryberg *et al.*, 2012). Another early *Pinus* from the Wealden Formation (*P. belgica*; Alvin, 1960) is not well dated because its exact stratigraphic position is unknown and the age of the Wealden varies considerably. We use *P. yorkshirensis* only in FBD analyses (as a stem member of the genus *Pinus*) because we did not include any outgroup species in this study; there is therefore no stem node to date in ND.

Subgenus *Pinus*

Pinus haboroensis Stockey et Nishida

Location: Haboro, Hokkaido
Age: Santonian – Maastrichtian, 85-65 Ma.

Pinus triphylla Hollick et Jeffrey

Location: Raritan and Magothy Formations, New York and Massachusetts, USA
Age: Turonian for Raritan, 90 Ma; Santonian to Campanian for Magothy, 85-70 Ma.

Discussion: Among extant *Pinus* there is a basic split between two clades: subgenus *Pinus* and subgenus *Strobus*. The presence of two vascular bundles in needles may be a synapormophy of subgenus *Pinus*, because extant *Pinus* outgroups (*Picea*, *Cathaya*) and members of subgenus *Strobus* generally have a single vascular bundle. Pine needles with two bundles become abundant in the Northern Hemisphere in the Late Cretaceous, with examples from the Coniacean-Santonian (89-83 Ma) of Georgia, USA, *Pinus triphylla* from the Raritan and Magothy Formations in the Northeastern United States, and the more poorly dated *Pinus haboroensis* from Japan (Stockey & Nishida, 1986). A split between subgenus *Strobus* and subgenus *Pinus* by the Late Cretaceous would also be consistent with evidence of subgenus *Strobus* based on wood anatomy (Xu *et al.*, 2015). In the primary analyses for this study, we therefore placed *P. triphylla* on the stem node of subgenus *Pinus* in ND. In FBD, we assigned both fossil taxa to the stem branch of subgenus *Pinus,* thus representing either a direct ancestor or an extinct tip along this branch.

Nevertheless, these needles cannot be readily placed in a phylogenetic analysis given their limited morphological and anatomical information. Moreover, traits that appear to be clear synapomorphies among extant taxa can be more ambiguous when compared to known fossil diversity. For example, *Pinus arnoldii* from the Eocene Princeton Chert (Klymiuk et al., 2011) exhibits a combination of traits characteristic of subgenus *Strobus* (needles with a single vascular bundle) and *Pinus* (densely sclerotic outer cone cortex),

suggesting that it can be difficult to definitely assign fossils to modern clades based on single organs or traits. Given these issues, and because age constraints on the split between subgenus *Strobus* and subgenus *Pinus* are likely to impact many other nodes in the tree, we ran separate analyses to bracket the possibility that these fossils either are or are not related to extant subgenus *Pinus* by removing them from the fossil calibration sets (AH 1 & 2).

Section *Trifoliae* subsection *Australes*

Pinus storeyana Axelrod

Location: Coal Valley Formation, Celetom Quarry, Reno, Nevada
Age: Late Miocene, 12 Ma

Discussion: This species shows robust and thickened apophyses on their seed cone scales, and these inflated apophyses preferentially occur on one side of the cone. Similar morphology is today found in some members of the *Attenuatae* subclade of subection *Australes* (*P. radiata, P. muricata, P. glabra*, and *P. attenuata*), particularly in *P. attenuata* and *P. muricata.* Asymmetrically inflated cone scales can occur in other pines; for example, the unrelated species *Pinus pseudostrobus* var. *apulcensis* also has asymmetrically thickened cones. The thickenings in this species are different than those of the *Attenuatae* and of the fossil cones, however; *Attenuatae* species have smooth pyramidal apophyses that project strongly but have relatively small umbos. *Pinus pseudostrobus* var. *apulcensis*, in contrast, has proportionally large and highly projecting umbos with smaller, more irregularly inflated apophyses. Coupled with the range of the fossils, which is either inside or just outside the Californian range of extant *Attenuatae* with inflated apophyses, we assign this fossil to the *Attenuatae* clade. It is difficult to determine if the fossil should be in crown *Attenuatae* or along the stem, because the two most similar species (*P. attenuata* and *P. muricata*) occur at the base of the clade and nested within it respectively. We therefore place it on the stem node in ND (split between *Attenuatae* and the rest of *Australes*), while in FBD we allow them to be placed anywhere within the clade. This fossil is included in all alternative hypotheses (AH 1-4).

Pinus radiata D. Don

Location: Santa Barbara Formation, Santa Barbara, California
Age: Pleistocene, ~400,000 years ago.

Discussion: These recent fossils (see Axelrod, 1986) appear very similar to modern *P. radiata* (large cones borne in clusters of 3 or 4 on a branch, with thickened apophyses near the base of the cone) and are also found in a similar geographic area to living members. In ND, we therefore used them to date the split between *P. radiata* and its sister species, *P. muricata*. In FBD, we allowed this species to be placed anywhere along the stem branch of extant *P. radiata.* These fossils are included in all alternative hypotheses (AH 1-4).

Section *Trifoliae* subsection *Ponderosae*

Pinus pieperi E. Dorf

Location: Mount Eden Flora, Beaumont, California
Age: Late Miocene, 5-6 Ma.

Discussion: This species is based on large isolated cone scales (originally described as *Pinus hazeni*) that have extremely thickened, curved, spine-like apophyses and umbos on their ovuliferous scales, similar to extant *P. sabiniana* or *P. coulteri* (Axelrod, 1986). This combination of features is characteristic of extant *P. sabiniana* and *P. coulteri* today, which belong to a subclade within the Ponderosae based on Parks et al. (2012), where (*P. coulteri + P. sabiniana + P. torreyana*) form a monophyletic sister clade to *P. jeffreyi.* In ND, we took a conservative approach and placed these fossil scales at the node of the MRCA of *P. sabianana* and *P. jeffreyi*. In FBD, we allowed it to be placed anywhere along the stem branch of the (*P. sabiniana + P. coulteri + P.torreyana*)-clade.

On the other hand, this species is based on highly fragmentary remains and its full morphology and anatomy are unknown. The assignment of these fossils to the modern subclade could be questioned due to our limited fossil information, and we therefore removed these fossils in three of the four additional hypotheses (AH 1-3) in order to test the potential effect of their inclusion on estimated ages.

Pinus truckeensis Axelrod

Location: Coal Valley Formation, Celetom Quarry, Reno, Nevada
Age: Late Miocene, 12 Ma.

Discussion: These well preserved cones were described as strongly resembling *P. pseudostrobus* (Axelrod, 1986), although members of subsection *Ponderosae* generally lack particularly diagnostic features. We therefore included this species only in the 'long list', in order to encompass the reasonable possibility that the *Ponderosae* subclade had evolved by the Late Miocene. In FBD, we allowed this fossil to be placed anywhere within section *Ponderosae* except within the *Sabinianae* subclade, as it lacks diagnostic features of this group (see *P. pieperi* above). We did not use this fossil in ND, as it is too difficult to justify its use without credible synapomorphies. We also removed this fossil from all the additional hypotheses (AH 1-4).

Pinus riogrande Axelrod

Location: Creede Flora, Creede, Colorado
Age: Late Oligocene, 27.2 Ma.

Discussion: This species suffers the same lack of diagnostic features as *P. truckeensis*, and its earlier age makes its use even more problematic. Wolfe and Schorn's generally critical revision of the Creede Flora (Wolfe & Schorn, 1990) retained this species in subsection *Ponderosae*, although they did not provide specific synapormorphies to justify this assignment beyond the general similarity of the seed cones. Such a lack of synapomorphies precludes its use in the small fossil set as well as in ND generally. However, in FBD we allowed the fossil to be placed anywhere within the subsection *Ponderosae*. We removed this fossil from all the additional hypotheses (AH 1-4) to test its potential effect on age estimates.

Section *Trifoliae* subsection *Contortae*

Pinus weasmaii Miller

Location: Glenns Ferry Formation, Idaho
Age: Pliocene, ~3 Ma.

Discussion: Miller (1992) compared this cone to modern *P. contorta* and found the cones to be virtually indistinguishable. They differ from closely related *P. banksiana* in continuous characters (e.g., size of the cones), which are not generally considered strong synapomorphies in fossil dating analyses. We therefore used this taxon only in the ‘long list’ of the FBD analysis and placed it along the stem of *P. contorta*. Given its morphological similarity to modern *P. contorta* and its young age, however, we felt there was a good chance these fossils were accurately placed. In order to test this potential effect, we kept this fossil in one of the alternative hypotheses (AH 4).

Section *Pinus*

Pinus baileyi Axelrod

Location: Thunder Mountain Flora, Idaho
Age: 45 Ma.

Discussion: Cones of *P. baileyi* have perexcentromucronate umbos, a term which refers to the position of the small spine, or mucro, in the umbo of the cone scale (Erwin & Schorn, 2006). This feature has traditionally been considered to be characteristic of section *Pinus* subsection *Pinus*, which more recent phylogenetic treatments have broken into two clades, subsection *Pinus* and subsection *Pinaster*, both of which have members with perexcentromucronate umbos as well as members with other umbo morphologies (Klaus, 1980). *P. baileyi* is considered most similar to the Asian species *P. kesiya* and *P. massoniana* in subsection *Pinus* (Erwin & Schorn, 2006), and differs from species with perexcentromucronate members in subsection *Pinaster* in that its umbos are protruding rather than flat. *Pinus baileyi* is also more similar to subsection *Pinus* in having inflated rhombic apophyses rather than rounded flat apophyses as in subsection *Pinaster*. This combination of traits may suggest that *P. baileyi* belongs in subsection *Pinus*, but the exact order of character evolution among these clades is unclear and thus the fossil cannot be placed with certainty. Given that perexcentromucronate umbos occur only in section *Pinus*, we placed this fossil on the stem node of this clade in ND. In FBD, we placed it along the stem branch of section *Pinus* subsection *Pinus*. This taxon is included in all alternative hypotheses (AH 1-4).

Section *Pinus* subsection *Pinus*

Pinus prekesiya Xing, Liu et Zhou

Location: Xiaolongtain Formation, Xianfeng Coalmine, Yunnan Province
Age: Upper Miocene, 11.6 - 5.3 Ma.

Discussion: These well-preserved cones were assigned to section *Pinus* subsection *Pinus* based on the presence of perexcentromucronate umbos (Xing *et al.*, 2010) and were further linked to extant *P. kesiya* based on their specific combination of characters: unlike some other perexcentromucronate pines of subsection *Pinus* (*P. resinosa, P. massoniana*), *P. prekesiya* has a vallum, or a protruding region of the apophysis, around

the umbo, and unlike perexcentromucronate pines of subsection *Pinaster*, it has protruding, rhomboidal apophyses with well-developed transverse keels and ridges. These features are also shared with extant *P. yunnanensis*, which is in a subclade of subsection *Pinus* along with *P. kesiya*. Given the age and geographic location of the fossils, it is reasonable to suggest they belong within this subclade. In ND, we place the fossil within the subclade suggested by the authors, defined by the MRCA of *P. yunnanensis, P. tabuliformis, P. kesiya,* and *P. densata*. In FBD the fossil is placed anywhere within the clade. On the other hand, the order of evolution of umbo characters in subsection *Pinus* is unclear. We therefore removed this fossil from three of the additional hypotheses (AH 1-3) in order to explore its effect on ages within the section.

Pinus fujiii (Yasui) Miki

Location: Multiple, Japan
Age: Lower to Upper Miocene, 15 Ma.

Discussion: These fossils are similar to some members of the Asian subclade within subsection *Pinus* mentioned above (MRCA of *P. yunnanensis* and *P. kesiya*) in having perexcentromucronate umbos with a well-defined vallum (Yamada *et al.*, 2014). On the other hand, the leaves of *P. fujiii* have resin canals that do not attach to the hypodermis of the leaf, which is more similar to extant *P. thunbergii*, a species that belongs to a subclade that is sister to the perexcentromucronate clade mentioned above. The combination of all these features suggests that the order of character evolution in these clades is not currently well understood, and that umbo and apophysis features may not be strong synapomorphies in general. When used for ND in the primary analyses, we treat it conservatively as a member of the broader Asian subclade of subsection *Pinus* (MRCA *P. taiwanensis* + *P. densata*), using the fossil to date the MRCA of this broader subclade. In FBD, we applied a less conservative placement and let it be placed anywhere along the stem of the (*P. yunnanensis - P. densata*.) subclade.

Given the uncertainties in interpreting the umbo characters and therefore the placement of these fossils, we removed them from three of the additional hypotheses (AH 1-3). To test the effect of a more conservative approach in FBD, we also let this fossil be placed anywhere within the broader subclade (MRCA *P. taiwanensis* + *P. densata*) in one additional hypothesis (AH 4).

Pinus premassoniana Su-Ting Ding et Bai-Nian Sun

Location: Shengxian Formation, Zhejiang, China
Age: Upper Miocene, 11.6-5.3 Ma

Discussion: This species was compared to extant *P. massoniana* based on needle anatomy and cone morphology (Ding *et al.*, 2013). Its ovuliferous scales have perexcentromucronate umbos, and as in previous fossils, this links it to either subsection *Pinus* or subsection *Pinaster*. As with other Miocene Chinese Pinaceae fossils, this species has more rhomboidal, protruding apophyses than extant perexcentromucronate species of subsection *Pinaster*. However, the umbos of *P. premassoniana* appear to lack a vallum, which potentially excludes it from the subclade of Asian perexcentromucronate pines described above (i.e., MRCA *P. yunnanensis* and *P. kesiya*). It therefore appears most similar to *P. massoniana* among extant species, and in ND we placed it on the stem node of extant *P. massoniana* while in FBD we let it be placed anywhere along the branch of extant *P. massoniana.* On the other hand, there are no strong synapomorphies or unique combinations of characters to supporting its assignment to

the extant *P. massoniana* lineage, so we used this fossil only in the 'long list' and we removed it in the additional analyses (AH 1-4).

Pinus densiflora Sieb. et Zucc.

Location: multiple
Age: 1.1 Ma.

Discussion: In their description of subsection *Pinus* fossils from Japan, Yamada *et al.* (2014) report *P. densiflora* fossils from as much as 2 Ma, although the provenance of this particular specimen is unclear. Here, we used the next oldest specimen from 1.1 Ma as evidence of the extant species. Assignment of this material to *P. densiflora* rather than the similar *P. tabuliformis* is based primarily on leaf anatomy; namely, resin canals that contact the hypodermis, a feature that does not occur in *P. tabuliformis*. Given the young age of these specimens and their morphological similarity to extant *P. densiflora*, we used these fossils in ND to date the divergence of the extant species. In FBD we placed it along the stem branch of this extant species. This taxon is included in all alternative hypotheses (AH 1-4).

Section *Pinus* subsection *Pinaster*

Pinus canariensis Klaus

Location: Styrian and Vienna Basins, Austria
Age: Middle to Upper Miocene, Styrian Basin, 16.3-12.8 Ma and Vienna Basin, ~9 Ma.

Discussion: Klaus (1989) described fossil cones that can be assigned to extant *P. canariensis* based on their morphological similarity to living specimens. Cone morphology of *P. canariensis* can be variable, however, and the species lacks particularly strong synapomorphies. We used these fossils as stem members of the *P. canariensis* + *P. roxburghii* clade and therefore used it to date the stem node of this clade in ND, while in FBD they can be placed anywhere along the stem branch. This taxon is included in all alternative hypotheses (AH 1-4).

Pinus halepensis Klaus

Location: Styrian Basin, Austria
Age: Badenian, Middle Miocene, 16.3-12.8 Ma.

Discussion: Klaus (1989) figures fossil cones from the Middle Miocene that look identical to cones of modern *P. halepensis*. In ND, we used them to date the split between *P. halepensis* and its sister, *P. brutia*. In FBD, we placed it along the branch of extant *P. halepensis*. This taxon is included in all alternative hypotheses (AH 1-4).

Subgenus *Strobus*

Section *Quinquefoliae* subsection *Strobus*

Pinus florissantii Axelrod

Location: Florissant, Colorado
Age: Early Oligocene, 34 Ma.

Discussion: This cone (Axelrod, 1986) displays terminal umbos on its ovuliferous scales, which is a strong synapomorphy of the subsection *Strobus* clade within section *Quinquefolia* of subgenus *Strobus*. In ND, we used this taxon to date the stem node of subsection *Strobus*. It is unclear whether the subsection *Krempfianae* is the direct sister of subsection *Strobus* (Gernandt *et al.*, 2005) or if it is a clade formed by subsection *Gerardianae* and subsection *Krempfianae* as in Parks *et al.* (2012). To define the monophyletic taxon set for ND, we followed Parks *et al.* (2012) and included conservatively all species of the three subsections. In FBD, we placed it along the stem branch of subsection *Strobus.* This taxon is included in all alternative hypotheses (AH 1-4).

Pinus delmarensis Axelrod

Location: Del Mar Formation, San Diego, California
Age: Middle Eocene, 47.8-38 Ma.

Discussion: This cone is poorly preserved but is extremely large and cylindrical (Axelrod, 1986), a combination of features found only in some members of subsection *Strobus* among extant species. In FBD, we therefore also allowed it to be placed along the stem branch of subsection *Strobus*. In ND, we did not use this fossil because we have more confidence in the affinities of *P. florissantii*, which is placed on the same node. This fossil is not included in any of the alternative hypotheses (AH 1-4).

Section *Parrya* subsection *Balfourianae*

Pinus crossii Knowlton

Location: Creede Flora, Colorado
Age: 27 Ma.

Discussion: This common and well-known pine species has angular apophyses and elongate, hook-like centromucronate umbos (see Wolfe & Schorn, 1990). This distinctive morphology is shared only with living members of the bristlecone clade (subsection *Balfourianae*), and we therefore treat this fossil as a stem member of this clade. In ND, we used it to date the split between it and its sister, *P. nelsonii*. In FBD, we placed it along the stem branch of subsection *Balfourianae.* This taxon is included in all alternative hypotheses (AH 1-4).

Section *Parrya* subsection *Cembroides*

Pinus sanjuanensis Axelrod

Location: Creede Flora, Colorado
Age: 27 Ma.

Discussion: This material was assigned to subsection *Cembroides* by (Axelrod, 1986). The figured specimen does not clearly show details of its morphology, but the seed cones of subsection *Cembroides* (pinyon pines) are distinctive in their small size, relatively few scales, and large seeds. Due to the lack of specific information for this cone, we only used it in the large fossil set. In ND, we placed it at the stem node of subsection *Cembroides* and in FBD we allowed it to be placed anywhere along the stem branch of subsection *Cembroides*. We removed this fossil in the additional hypotheses (AH 1-4).

Pinus lindgrenii Knowlton

Location: Chalk Hills Formation, Idaho
Age: 6-7 Ma.

Discussion: These cones (Knowlton, 1901) undoubtedly belong to subsection *Cembroides*; they have relatively small cones with very large, wingless seeds. However, two clades within subsection *Cembroides* appear to have independently evolved this morphology and it is not clear where these cones belong. Axelrod linked them with the *P. edulis* clade (1986) because they have a relatively low apophysis, while cones of the *P. monophylla* clade have more inflated apophyses. However, Miller (1992) describes this character as too variable to be useful. In the large fossil set, we follow Axelrod's assessment (1986) and used this fossil to date the crown node of the *P. edulis* clade in ND, while in FBD we allowed it to be placed anywhere within the *P. edulis* clade. However, we acknowledged for a more conservative taxonomic assignment by placing these fossils within the whole subsection *Cembroides* in the additional hypotheses (AH 1-4).

References

Alvin, K.L. (1960) Further conifers of the Pinaceae from the Wealden Formation of Belgium. *Mémoires Institut Royal des Sciences Naturelles de Belgique*, **146**, 16-21.

Axelrod, D.I. (1986) Cenozoic history of some western American pines. *Annals of the Missouri Botanical Garden*, **73**, 565-641.

Axelrod, D.I. & Cota, J. (1993) A further contribution to closed-cone pine (Oocarpae) history. *American Journal of Botany*, **80**, 743-751.

Ding, S.T., Wu, J.Y., Chen, J.L., Yang, Y., Yan, D.F. & Sun, B.N. (2013) Needles and seed cones of *Pinus premassoniana* sp nov., and associated pollen cone from the upper Miocene in East China. *Review of Palaeobotany and Palynology*, **197**, 78-89.

Erwin, D.M. & Schorn, H.E. (2006) Pinus baileyi (section Pinus, Pinaceae) from the Paleogene of Idaho, USA. *American Journal of Botany*, **93**, 197-205.

Gernandt, D.S., Lopez, G.G., Garcia, S.O. & Liston, A. (2005) Phylogeny and classification of *Pinus*. *Taxon*, **54**, 29-42.

Klaus, W. (1980) Ein *Pinus canariensis* Smith-Zapfenfund aus dem Ober-Miozän (Pannon) des Wiener Beckens: A fossil cone of Pinus canariensis Smith from the Upper Miocene (Pannon) of the Vienna Basin (Austria). *Annalen des Naturhistorischen Museums in Wien. Serie A für Mineralogie und Petrographie, Geologie und Paläontologie, Anthropologie und Prähistorie*, **84**, 79-84.

Klaus, W. (1989) Mediterranean Pines and Their History. *Plant Systematics and Evolution*, **162**, 133-163.

Klymiuk, A.A., Stockey, R.A. & Rothwell, G.W. (2011) The first organismal concept for an extinct species of Pinaceae: *Pinus arnoldii* Miller. *International Journal of Plant Sciences*, **172**, 294-313.

Knowlton, F.H. (1901) A fossil nut pine from Idaho. *Torreya*, **1**, 113-115.

Miller, C.N. (1992) Structurally preserved cones of *Pinus* from the Neogene of Idaho and Oregon. *International Journal of Plant Sciences*, **153**, 147-154.

Parks, M., Cronn, R. & Liston, A. (2012) Separating the wheat from the chaff: mitigating the effects of noise in a plastome phylogenomic data set from *Pinus* L. (Pinaceae). *BMC Evolutionary Biology*, **12**, 100.

Robison, C.R. (1977) *Pinus triphylla* and *Pinus quinquefolia* from the Upper Cretaceous of Massachusetts. *American Journal of Botany*, **64**, 726-732.

Ryberg, P.E., Rothwell, G.W., Stockey, R.A., Hilton, J., Mapes, G. & Riding, J.B. (2012) Reconsidering relationships among stem and crown group Pinaceae: oldest record of the genus *Pinus* from the Early Cretaceous of Yorkshire, United Kingdom. *International Journal of Plant Sciences*, **173**, 917-932.

Smith, S.Y. & Stockey, R.A. (2001) A new species of *Pityostrobus* from the lower Cretaceous of California and its bearing on the evolution of Pinaceae. *International Journal of Plant Sciences*, **162**, 669-681.

Stockey, R.A. & Nishida, M. (1986) *Pinus haboroensis* sp.nov. and the affinities of permineralized leaves from the Upper Cretaceous of Japan. *Canadian Journal of Botany*, **64**, 1856-1866.

Wolfe, J.A. & Schorn, H.R. (1990) Taxonomic revision of the Spermatopsida of the Oligocene Creede flora, southern Colorado. *United States Geological Survey Bulletin 1923*, , 1-40.

Xing, Y.W., Liu, Y.S., Su, T., Jacques, F.M.B. & Zhou, Z.K. (2010) *Pinus prekesiya* sp nov from the upper Miocene of Yunnan, southwestern China and its biogeographical implications. *Review of Palaeobotany and Palynology*, **160**, 1-9.

Xu, Q.Q., Zhou, W.J., Kodrul, T.M., Naugolnykh, S.V. & Jin, J.H. (2015) Late Eocene white pines (*Pinus* subgenus Strobus) from southern China. *Scientific Reports*, **5**

Yamada, T., Yamada, M. & Tsukagoshi, M. (2014) Fossil records of subsection Pinus (genus *Pinus*, Pinaceae) from the Cenozoic in Japan. *Journal of Plant Research*, **127**, 193-208.
