# Supplementary material for: Fossils matter: improved estimates of divergence times in Pinus reveal older diversification
Source: BMC Evol Biol. 2017 Apr 4;17:95. doi: 10.1186/s12862-017-0941-z (PMC5381128; doi:10.1186/s12862-017-0941-z)
Supplement: Supplementary file 8 — Accession numbers of used gene sequences downloaded from GenBank. Asterisks on accession numbers indicate sequences that are not linked to a published journal article. (PDF 67 kb) [file 12862_2017_941_MOESM8_ESM.pdf]

Taxa	GenBank Accession numbers of chloroplast genes							
	accD	*matK*	*rbcL*	*rpl20*	*rpoB*	*rpoC1*	*trnV*	*ycf1*
Pinus albicaulis	FJ899566	FJ899566	FJ899566	FJ899566	FJ899566	FJ899566	FJ899566	FJ899566
P. amamiana	JN854226	JN854226	JN854226	JN854226	JN854226	JN854226	JN854226	JN854226
P. aristata	FJ899567	FJ899567	FJ899567	FJ899567	FJ899567	FJ899567	FJ899567	FJ899567
P. arizonica	JN854225	JN854225	JN854225	JN854225	JN854225	JN854225	JN854225	KC157172
P. armandii	FJ899568	AB161002*	FJ899568	FJ899568	FJ899568	FJ899568	FJ899568	FJ899568
P. attenuata	FJ899569	FJ899569	FJ899569	FJ899569	FJ899569	FJ899569	FJ899569	KC157112
P. ayacahuite	FJ899570	AY497257	FJ899570	FJ899570	FJ899570	FJ899570	FJ899570	FJ899570
P. balfouriana	-	AY115799	AY115760	-	-	-	AB019880	-
P. banksiana	FJ899571	EF440517	FJ899571	FJ899571	FJ899571	FJ899571	FJ899571	KC157149
P. bhutanica	-	AY497262	DQ353719*	-	-	-	-	-
P. brutia	JN854224	JN854224	JN854224	JN854224	JN854224	JN854224	JN854224	JN854224
P. bungeana	JN854223	JN854223	JN854223	JN854223	JN854223	JN854223	JN854223	JN854223
P. canariensis	FJ899572	AB084494*	FJ899572	FJ899572	FJ899572	FJ899572	FJ899572	FJ899572
P. caribaea	JN854222	JN854222	JN854222	JN854222	JN854222	JN854222	JN854222	JN854222
P. cembra	FJ899574	AB160985*	FJ899574	FJ899574	FJ899574	FJ899574	FJ899574	-
P. cembroides	JN854220	JN854220	JN854220	JN854220	JN854220	JN854220	JN854220	JN854220
P. chiapensis	JN854219	JN854219	JN854219	JN854219	JN854219	JN854219	JN854219	JN854219
P. clausa	JN854217	JN854217	JN854217	JN854217	JN854217	JN854217	JN854217	KC157189
P. contorta	EU998740	AY497266	EU998740	EU998740	EU998740	EU998740	EU998740	EU998740
P. cooperi	-	DQ353708*	DQ353723*	-	-	-	-	FJ580193
P. coulteri	JN854215	JN854215	JN854215	JN854215	JN854215	JN854215	JN854215	KC157139
P. cubensis	JN854214	JN854214	JN854214	JN854214	JN854214	JN854214	JN854214	KC157114
P. culminicola	JN854213	JN854213	JN854213	JN854213	JN854213	JN854213	JN854213	JN854213
P. dalatensis	JN854211	JN854211	JN854211	JN854211	JN854211	JN854211	JN854211	JN854211
P. densata	JN854209	JN854209	JN854209	JN854209	JN854209	JN854209	JN854209	JN854209
P. densiflora	JN854210	JN854210	JN854210	JN854210	JN854210	JN854210	JN854210	JN854210
P. devoniana	JN854208	JN854208	JN854208	JN854208	JN854208	JN854208	JN854208	KC157179
P. discolor	JN854207	JN854207	JN854207	JN854207	JN854207	JN854207	JN854207	JN854207
P. douglasiana	JN854205	JN854205	JN854205	JN854205	JN854205	JN854205	JN854205	JN854205
P. durangensis	-	AY497276	AY497240	-	-	-	DQ159460	KC157175
P. echinata	JN854204	JN854204	JN854204	JN854204	JN854204	JN854204	JN854204	KC157152
P. edulis	JN854203	JN854203	JN854203	JN854203	JN854203	JN854203	JN854203	JN854203
P. elliottii	JN854202	JN854202	JN854202	JN854202	JN854202	JN854202	JN854202	KC157123
P. engelmannii	JN854201	JN854201	JN854201	JN854201	JN854201	JN854201	JN854201	JN854201
P. fenzeliana	JN854212	JN854212	JN854212	JN854212	JN854212	JN854212	JN854212	-
P. flexilis	FJ899576	AY497258	FJ899576	FJ899576	FJ899576	FJ899576	FJ899576	FJ899576
P. fragilissima	JN854200	JN854200	JN854200	JN854200	JN854200	JN854200	JN854200.1	JN854200
P. gerardiana	EU998741	EU998741	EU998741	EU998741	EU998741	EU998741	EU998741	EU998741
P. glabra	JN854199	JN854199	JN854199	JN854199	JN854199	JN854199	JN854199	KC157154
P. greggii	JN854198	JN854198	JN854198	JN854198	JN854198	JN854198	JN854198	KC157126
P. halepensis	JN854197	JN854197	JN854197	JN854197	JN854197	JN854197	JN854197	JN854197
P. hartwegii	JN854206	JN854206	JN854206	JN854206	JN854206	JN854206	JN854206	KC157178
P. heldreichii	JN854195	JN854195	JN854195	JN854195	JN854195	JN854195	JN854195	JN854195
P. herrerae	-	AB080943*	AB063386*	-	AM883768*		AB063602*	KC157155
P. hwangshanensis	JN854194	JN854194	JN854194	JN854194	JN854194	JN854194	JN854194	JN854194
P. jaliscana	-	-	-	-	AM883769*	-	-	KC157194
P. jeffreyi	JN854193	JN854193	JN854193	JN854193	JN854193	JN854193	JN854193	KC157181
P. johannis	JN854192	JN854192	JN854192	JN854192	JN854192	JN854192	JN854192	JN854192

P. juarezensis	-	AY115770	AY115742	-	-	-	-	-
P. kesiya	JN854191	JN854191	JN854191	JN854191	JN854191	JN854191	JN854191	JN854191
P. koraiensis	AY228468*	AY228468*	AY228468*	AY228468*	AY228468*	AY228468*	AY228468*	AY228468*
P. krempfii	EU998742	EU998742	EU998742	EU998742	EU998742	EU998742	EU998742	EU998742
P. kwangtungensis	JN854153	JN854153	JN854153	JN854153	JN854153	JN854153	JN854153	JN854151
P. lambertiana	EU998743	EU998743	EU998743	EU998743	EU998743	EU998743	EU998743	EU998743
P. latteri	JN854190	JN854190	JN854190	JN854190	JN854190	JN854190	JN854190	JN854190
P. lawsonii	JN854188	JN854188	JN854188	JN854188	JN854188	JN854188	JN854188	KC157094
P. leiophylla	JN854218	JN854218	JN854218	JN854218	JN854218	JN854218	JN854218	KC157132
P. longaeva	-	EU998744	EU998744	EU998744	EU998744	EU998744	EU998744	EU998744
P. luchuensis	-	AB097780*	AB097772*	-	-	-	AB097788*	-
P. lumholtzii	JN854186	JN854186	JN854186	JN854186	JN854186	JN854186	JN854186	KC157168
P. maestrensis	-	AB080939*	AB063371*	-	-	-	AB063587*	-
P. massoniana	JN854185	JN854185	JN854185	JN854185	JN854185	JN854185	JN854185	KC427272
P. maximartinezii	JN854184	JN854184	JN854184	JN854184	JN854184	JN854184	JN854184	JN854184
P. maximinoi	-	AB161010*	AB161040*	-	AM883786*	-	-	KC157109
P. merkusii	FJ899579	AY497287	AY497251	-	FJ899579	FJ899579	FJ899579	FJ899579
P. monophylla	-	EU998745	EU998745	EU998745	EU998745	EU998745	EU998745	EU998745
P. montezumae	JN854183	JN854183	JN854183	JN854183	JN854183	JN854183	JN854183	JN854183
P. monticola	FJ899580	GQ478182*	AY497223	GQ478185*	FJ899580	FJ899580	FJ899580	FJ899580
P. morrisonicola	JN854182	JN854182	JN854182	JN854182	JN854182	JN854182	JN854182	JN854182
P. mugo	JN854181	JN854181	JN854181	JN854181	JN854181	JN854181	JN854181	JN854181
P. muricata	JN854180	AB080935*	JN854180	JN854180	JN854180	JN854180	JN854180	JN854180
P. nelsonii	EU998746	EU998746	EU998746	EU998746	EU998746	EU998746	EU998746	EU998746
P. nigra	JN854179	JN854179	JN854179	JN854179	JN854179	JN854179	JN854179	JN854179
P. occidentalis	JN854177	JN854177	JN854177	JN854177	JN854177	JN854177	JN854177	JN854177
P. oocarpa	-	AB081084*	DQ353726*	-	AM883774*	-	AB063598*	KC157141
P. palustris	JN854176	JN854176	JN854176	JN854176	JN854176	JN854176	JN854176	KC157163
P. parviflora	FJ899581	FJ899581	EU269033	FJ899581	FJ899581	FJ899581	AB019874	FJ899581
P. patula	JN854175	JN854175	JN854175	JN854175	JN854175	JN854175	JN854175	JN854175
P. peuce	FJ899582	AY497254	FJ899582	FJ899582	FJ899582	FJ899582	FJ899582	FJ899582
P. pinaster	FJ899583	FJ899583	FJ899583	FJ899583	FJ899583	FJ899583	FJ899583	FJ899583
P. pinceana	JN854174	JN854174	JN854174	JN854174	JN854174	JN854174	JN854174	JN854174
P. pinea	JN854173	JN854173	JN854173	JN854173	JN854173	JN854173	JN854173	JN854173
P. ponderosa	JN854172	JN854172	JN854172	JN854172	JN854172	JN854172	JN854172	KC157127
P. praetermissa	-	DQ353711*	DQ353727*	-	-	-	-	KC157122
P. pringlei	JN854189	JN854189	JN854189	JN854189	JN854189	JN854189	JN854189	KC157095
P. pseudostrobus	JN854178	JN854178	JN854178	JN854178	JN854178	JN854178	JN854178	KC157192
P. pumila	JN854168	JN854168	JN854168	JN854168	JN854168	JN854168	JN854168	-
P. pungens	JN854167	JN854167	JN854167	JN854167	JN854167	JN854167	JN854167	KC157206
P. quadrifolia	JN854166	JN854166	JN854166	JN854166	JN854166	JN854166	JN854166	JN854166
P. radiata	JN854165	JN854165	JN854165	JN854165	JN854165	JN854165	JN854165	KC157207
P. remota	JN854164	JN854164	JN854164	JN854164	JN854164	JN854164	JN854164	JN854164
P. resinosa	FJ899556	FJ899556	FJ899556	FJ899556	FJ899556	FJ899556	FJ899556	KC157078
P. rigida	JN854163	JN854163	JN854163	JN854163	JN854163	JN854163	JN854163	KC157079
P. roxburghii	JN854162	JN854162	JN854162	JN854162	JN854162	JN854162	JN854162	JN854162
P. rzedowskii	FJ899557	FJ899557	FJ899557	FJ899557	FJ899557	FJ899557	-	FJ899557
P. sabiniana	JN854161	JN854161	JN854161	JN854161	JN854161	JN854161	JN854161	KC157111
P. serotina	JN854160	JN854160	JN854160	JN854160	JN854160	JN854160	JN854160	KC157164
P. sibirica	FJ899558	FJ899558	FJ899558	FJ899558	FJ899558	FJ899558	FJ899558	FJ899558
P. squamata	FJ899559	FJ899559	FJ899559	FJ899559	FJ899559	FJ899559	FJ899559	FJ899559
P. strobiformis	JN854159	JN854159	JN854159	JN854159	JN854159	JN854159	JN854159	JN854159
P. strobus	FJ899560	FJ899560	FJ899560	FJ899560	FJ899560	FJ899560	FJ899560	FJ899560

P. sylvestris	JN854158	JN854158	JN854158	JN854158	JN854158	JN854158	JN854158	JN854158
P. tabuliformis	-	AB161015*	AY555717	-	AM883727*	-	AB019884	-
P. taeda	FJ899561	FJ899561	FJ899561	FJ899561	FJ899561	FJ899561	FJ899561	KC157197
P. taiwanensis	JN854157	JN854157	JN854157	JN854157	JN854157	JN854157	JN854157	JN854157
P. tecunumanii	-	-	-	-	-	-	-	KC157187
P. teocote	-	AB097783*	AB097773*	-	-	-	AB097789*	KC157202
P. thunbergii	FJ899562	FJ899562	FJ899562	FJ899562	FJ899562	FJ899562	FJ899562	FJ899562
P. torreyana	FJ899564	AY497273	FJ899564	FJ899564	FJ899564	FJ899564	FJ899564	KC157182
P. tropicalis	JN854156	JN854156	JN854156	JN854156	JN854156	JN854156	JN854156	JN854156
P. virginiana	JN854155	AB080923*	JN854155	JN854155	JN854155	JN854155	JN854155	KC157196
P. wallichiana	JN854154	JN854154	JN854154	JN854154	JN854154	JN854154	JN854154	JN854154
P. washoensis	-	DQ353706*	DQ353721*	-	-	-	DQ159463	-
P. yecorensis	JN854152	JN854152	JN854152	JN854152	JN854152	JN854152	JN854152	KC157128
P. yunnanensis	JN854151	JN854151	JN854151	JN854151	JN854151	JN854151	JN854151	JN854151
