# Supplementary material for: Fossils matter: improved estimates of divergence times in Pinus reveal older diversification
Source: BMC Evol Biol. 2017 Apr 4;17:95. doi: 10.1186/s12862-017-0941-z (PMC5381128; doi:10.1186/s12862-017-0941-z)
Supplement: Supplementary file 9 — Sequence matrix used for phylogenetic inference. The number of nucleotide base pairs per gene sequence used for each pine species. N in parentheses gives the number of positions in this gene sequence for which the nucleotide pair is undetermined. (PDF 68 kb) [file 12862_2017_941_MOESM9_ESM.pdf]

Taxon	Total length (bp)	bp (%)	Char-sets	Chloroplast gene							
				accD	*matK*	*rbcL*	*rpl20*	*rpoB*	*rpoC1*	*trnV*	*ycf1*
Pinus albicaulis	5866	100	8	910	1380	1254	95	343	383	482	1019
P. amamiana	5866	100	8	910	1380	1254 (1N)	95	343	383	482	1019 (1N)
P. aristata	5866	100	8	910	1380	1254	95	343	383	482	1019
P. arizonica	5866	100	8	910	1380	1254	95	343	383	482	1019
P. armandii	5866	100	8	910	1380	1254	95	343	383	482	1019
P. attenuata	5866	100	8	910	1380	1254	95	343	383	482	1019
P. ayacahuite	5866	53	8	910	1380	1254	95	343	383	482	1019
P. balfouriana	3116	100	3	-	1380	1254	-	-	-	482	-
P. banksiana	5866	45	8	910	1380	1254	95	343	383	482	1019
P. bhutanica	2634	100	2	-	1380	1254	-	-	-	-	-
P. brutia	5866	100	8	910	1380	1254	95	343	383	482	1019
P. bungeana	5866	92	8	910	1380 (2N)	1254	95	343	383	482	1019 (1N)
P. canariensis	5384	100	7	910	1380	1254	95	343	383	-	1019
P. caribaea	5866	83	8	910	1380	1254	95	343	383	482	1019
P. cembra	4847	100	7	910	1380	1254	95	343	383	482	-
P. cembroides	5866	100	8	910	1380	1254	95	343	383	482	1019
P. chiapensis	5866	100	8	910	1380 (2N)	1254	95	343	383	482	1019
P. clausa	5866	100	8	910	1380	1254	95	343	383	482	1019
P. contorta	5866	45	8	910	1380	1254	95	343	383	482	1019
P. cooperi	2634	100	2	-	1380	1254	-	-	-	-	-
P. coulteri	5866	100	8	910	1380	1254	95	343	383	482	1019
P. cubensis	5866	100	8	910	1380	1254	95	343	383	482	1019
P. culminicola	5866	100	8	910	1380	1254	95	343	383	482	1019
P. dalatensis	5866	83	8	910 (29N)	1380 (132N)	1254	95	343	383	482	1019
P. densata	4847	100	7	910	1380	1254	95	343	383	482	-
P. densiflora	5866	100	8	910	1380	1254	95	343	383	482	1019
P. devoniana	5866	83	8	910	1380	1254	95	343	383	482	1019
P. discolor	4847	100	7	910	1380	1254	95	343	383	482	-
P. douglasiana	5866	70	8	910	1380	1254	95	343	383	482	1019 (2N)
P. durangensis	4135	100	4	-	1380	1254	-	-	-	482	1019
P. echinata	5866	100	8	910	1380	1254	95	343	383	482	1019
P. edulis	5866	100	8	910	1380 (3N)	1254	95	343	383	482	1019 (7N)
P. elliottii	5866	100	8	910	1380	1254	95	343	383	482	1019
P. engelmannii	5866	83	8	910	1380	1254	95	343	383	482	1019
P. fenzeliana	4847	100	7	910 (3N)	1380	1254	95	343 (145N)	383	482	-
P. flexilis	5866	100	8	910	1380	1254	95	343	383	482	1019
P. fragilissima	5866	93	8	910	1380 (28N)	1254	95	343	383	482	1019
P. gerardiana	5428	100	6	910	1380	1254	-	-	383	482	1019
P. glabra	5866	100	8	910 (6N)	1380 (2N)	1254	95	343	383	482	1019
P. greggii	5866	100	8	910	1380	1254	95	343	383	482	1019
P. halepensis	5866	100	8	910	1380	1254	95	343	383 (1N)	482	1019
P. hartwegii	5866	100	8	910	1380	1254	95	343	383	482	1019
P. heldreichii	5866	76	8	910	1380	1254	95	343 (1N)	383 (27N)	482	1019 (8N)
P. herrerae	4478	100	5	-	1380	1254	-	343	-	482	1019
P. hwangshanensis	5866	23	8	910	1380	1254	95	343	383	482	1019
P. jaliscana	1362	100	2	-	-	-	-	343	-	-	1019
P. jeffreyi	5866	100	8	910	1380	1254	95	343	383	482	1019
P. johannis	5866	45	8	910 (1N)	1380	1254	95	343	383	482	1019
P. juarezensis	2634	100	2	-	1380	1254	-	-	-	-	-
P. kesiya	5866	100	8	910	1380	1254	95	343	383	482	1019
P. koraiensis	5866	100	8	910	1380	1254	95	343	383	482	1019
P. krempfii	5866	100	8	910	1380	1254	95	343	383	482	1019
P. kwangtungensis	5866	100	8	910	1380 (31N)	1254	95	343	383	482	1019 (12N)
P. lambertiana	5866	100	8	910	1380	1254	95	343	383	482	1019
P. latteri	5866	100	8	910	1380 (2N)	1254	95	343	383	482	1019
P. lawsonii	5866	100	8	910	1380	1254	95	343	383	482	1019
P. leiophylla	5866	84	8	910	1380	1254	95	343	383	482	1019
P. longaeva	4956	53	7	-	1380	1254	95	343	383	482	1019
P. luchuensis	3116	100	3	-	1380	1254	-	-	-	482	-
P. lumholtzii	5866	53	8	910	1380	1254	95	343	383	482	1019
P. maestrensis	3116	100	3	-	1380	1254	-	-	-	482	-
P. massoniana	5866	100	8	910	1380	1254	95	343	383	482	1019
P. maximartinezii	5866	68	8	910	1380	1254	95	343	383	482	1019
P. maximinoi	3996	98	4	-	1380	1254	-	343	-	-	1019
P. merkusii	5771	84	7	910	1380	1254	-	343	383	482	1019
P. monophylla	4956	100	7	-	1380	1254	95	343	383	482	1019
P. montezumae	5866	100	8	910	1380	1254	95	343	383	482	1019
P. monticola	5866	100	8	910	1380	1254	95	343	383	482	1019
P. morrisonicola	5866	100	8	910	1380	1254	95	343	383	482	1019 (6N)
P. mugo	5866	100	8	910	1380	1254	95	343	383	482	1019
P. muricata	5866	100	8	910	1380	1254	95	343	383	482	1019
P. nelsonii	5866	100	8	910	1380	1254	95	343	383	482	1019
P. nigra	5866	100	8	910	1380	1254	95	343	383	482	1019
P. occidentalis	5866	100	8	910	1380	1254	95	343	383	482	1019
P. oocarpa	4478	76	5	-	1380	1254 (1N)	-	343	-	482	1019
P. palustris	5866	100	8	910	1380	1254	95	343	383	482	1019
P. parviflora	5866	100	8	910	1380	1254	95	343	383	482	1019
P. patula	5866	100	8	910	1380	1254	95	343	383	482	1019
P. peuce	5866	100	8	910	1380	1254	95	343	383	482	1019
P. pinaster	5866	100	8	910	1380	1254	95	343	383	482	1019
P. pinceana	5866	100	8	910 (2N)	1380	1254	95	343	383	482	1019
P. pinea	5866	100	8	910 (2N)	1380	1254	95	343	383	482	1019
P. ponderosa	5866	100	8	910	1380	1254	95	343	383	482	1019
P. praetermissa	3653	62	3	-	1380	1254	-	-	-	-	1019
P. pringlei	5866	100	8	910	1380	1254	95	343	383 (1N)	482	1019
P. pseudostrobus	5866	100	8	910 (4N)	1380 (3N)	1254	95	343 (50N)	383	482	1019
P. pumila	4847	83	7	910	1380	1254	95	343	383	482	-
P. pungens	5866	100	8	910	1380	1254	95	343	383	482	1019
P. quadrifolia	5866	100	8	910	1380	1254	95	343	383	482	1019
P. radiata	5866	100	8	910	1380	1254	95	343	383	482	1019
P. remota	5866	100	8	910	1380	1254	95	343	383	482	1019

P. resinosa	5866	100	8	910	1380	1254	95	343	383	482	1019
P. rigida	5866	100	8	910	1380	1254	95	343	383	482	1019
P. roxburghii	5866	100	8	910	1380	1254	95	343	383	482	1019
P. rzedowskii	5384	92	7	910	1380	1254	95	343	383	-	1019
P. sabiniana	5866	100	8	910	1380	1254	95	343	383	482	1019
P. serotina	5866	100	8	910	1380	1254	95	343	383	482	1019
P. sibirica	5771	98	7	910	1380	1254	-	343	383	482	1019
P. squamata	5866	100	8	910	1380	1254	95	343	383	482	1019
P. strobiformis	5866	100	8	910	1380	1254	95	343	383	482	1019
P. strobus	5771	98	7	910	1380	1254	-	343	383	482	1019
P. sylvestris	5866	100	8	910	1380	1254	95	343	383	482	1019
P. tabuliformis	3459	59	4	-	1380	1254	-	343	-	482	-
P. taeda	5866	100	8	910	1380	1254	95	343	383	482	1019
P. taiwanensis	4847	83	7	910	1380	1254	95	343 (24N)	383	482	-
P. tecunumanii	1019	17	1	-	-	-	-	-	-	-	1019
P. teocote	4135	70	4	-	1380	1254	-	-	-	482	1019
P. thunbergii	5866	100	8	910	1380	1254	95	343	383	482	1019
P. torreyana	5866	100	8	910	1380	1254	95	343	383	482	1019
P. tropicalis	5866	100	8	910	1380	1254	95	343 (2N)	383	482	1019 (28N)
P. virginiana	5866	100	8	910 (1N)	1380	1254	95	343 (142N)	383	482	1019
P. wallichiana	5866	100	8	910	1380	1254	95	343	383	482	1019 (1N)
P. washoensis	3116	53	3	-	1380	1254	-	-	-	482	-
P. yecorensis	5866	100	8	910	1380	1254	95	343	383	482	1019
P. yunnanensis	5866	100	8	910	1380	1254	95	343 (7N)	383 (23N)	482	1019 (34N)
