# Supplementary material for: Fossils matter: improved estimates of divergence times in Pinus reveal older diversification
Source: BMC Evol Biol. 2017 Apr 4;17:95. doi: 10.1186/s12862-017-0941-z (PMC5381128; doi:10.1186/s12862-017-0941-z)
Supplement: Supplementary file 10 — Settings and output summary of PartitionFinder showing the best partition scheme for the used sequence data in this study. (PDF 30 kb) [file 12862_2017_941_MOESM10_ESM.pdf]

Settings used

alignment : ./chloro_all_spp_PARTITIONFINDER.nex

branchlengths : linked

models : HKY+G, GTR+I, GTR+G, HKY+I, GTR, HKY, JC+G, JC+I, JC

model_selection : bic

search : greedy

Best partitioning scheme

Scheme Name : step_5

Scheme lnL : -15786.16616

Scheme BIC : 33758.9182389

Number of params : 252

Number of sites : 5866

Number of subsets : 3

Subset	Best Model	Subset Partitions	Subset Sites
1	GTR+I	accD_fasta, matK_fasta, rpoB_fasta, rpoC1_fasta, trnV_fasta	1-910, 911-2290, 3640-3982, 3983-4365, 4366-4847
2	HKY+I	rbcL_fasta, rpl20_fasta	2291-3544, 3545-3639
3	GTR+G	ycf1_fasta	4848-5866

Scheme Description in PartitionFinder format

Scheme_step_5 = (accD_fasta, matK_fasta, rpoB_fasta, rpoC1_fasta, trnV_fasta) (rbcL_fasta, rpl20_fasta) (ycf1_fasta);

RaxML-style partition definitions

DNA, p1 = 1-910, 911-2290, 3640-3982, 3983-4365, 4366-4847

DNA, p2 = 2291-3544, 3545-3639

DNA, p3 = 4848-5866
